# Regulation of ROS Metabolism in Plants under Environmental Stress: A Review of Recent Experimental Evidence

**DOI:** 10.3390/ijms21228695

**Published:** 2020-11-18

**Authors:** Mirza Hasanuzzaman, M. H. M. Borhannuddin Bhuyan, Khursheda Parvin, Tasnim Farha Bhuiyan, Taufika Islam Anee, Kamrun Nahar, Md. Shahadat Hossen, Faisal Zulfiqar, Md. Mahabub Alam, Masayuki Fujita

**Affiliations:** 1 Department of Agronomy, Faculty of Agriculture, Sher-e-Bangla Agricultural University, Sher-e-Bangla Nagar, Dhaka 1207, Bangladesh; taufiqaislam@gmail.com (T.I.A.); shamim1983@yahoo.com (M.M.A.); 2 Citrus Research Station, Bangladesh Agricultural Research Institute, Jaintapur, Sylhet 3156, Bangladesh; razon_sau@yahoo.com; 3 Laboratory of Plant Stress Responses, Faculty of Agriculture, Kagawa University, Miki-cho, Kita-Gun, Kagawa 761-0795, Japan; hirasau@gmail.com; 4 Department of Horticulture, Faculty of Agriculture, Sher-e-Bangla Agricultural University, Sher-e-Bangla Nagar, Dhaka 1207, Bangladesh; 5 Department of Agricultural Botany, Faculty of Agriculture, Sher-e-Bangla Agricultural University, Sher-e-Bangla Nagar, Dhaka 1207, Bangladesh; farhatasnim28@gmail.com (T.F.B.); knahar84@yahoo.com (K.N.); 6 Independent Researcher, Dhaka 1207, Bangladesh; shahadat.hossen32@yahoo.com; 7 Institute of Horticultural Sciences, Faculty of Agriculture, University of Agriculture, Faisalabad 38000, Pakistan; ch.faisal.zulfiqar@gmail.com

**Keywords:** abiotic stress, reactive oxygen species, oxidative stress, antioxidant defense system, abiotic stress tolerance

## Abstract

Various environmental stresses singly or in combination generate excess amounts of reactive oxygen species (ROS), leading to oxidative stress and impaired redox homeostasis. Generation of ROS is the obvious outcome of abiotic stresses and is gaining importance not only for their ubiquitous generation and subsequent damaging effects in plants but also for their diversified roles in signaling cascade, affecting other biomolecules, hormones concerning growth, development, or regulation of stress tolerance. Therefore, a good balance between ROS generation and the antioxidant defense system protects photosynthetic machinery, maintains membrane integrity, and prevents damage to nucleic acids and proteins. Notably, the antioxidant defense system not only scavenges ROS but also regulates the ROS titer for signaling. A glut of studies have been executed over the last few decades to discover the pattern of ROS generation and ROS scavenging. Reports suggested a sharp threshold level of ROS for being beneficial or toxic, depending on the plant species, their growth stages, types of abiotic stresses, stress intensity, and duration. Approaches towards enhancing the antioxidant defense in plants is one of the vital areas of research for plant biologists. Therefore, in this review, we accumulated and discussed the physicochemical basis of ROS production, cellular compartment-specific ROS generation pathways, and their possible distressing effects. Moreover, the function of the antioxidant defense system for detoxification and homeostasis of ROS for maximizing defense is also discussed in light of the latest research endeavors and experimental evidence.

## 1. Introduction

Ensuring food security is one of the most defined concerns and high-priority areas among plant scientists [1]. Meanwhile, agricultural productivity is under immense pressure in the scenario of stringent climate change and an ever-increasing affluent population [2]. Under such a scenario, it is imperative to understand the coordinated processes of plant metabolism to improve crop productivity. Due to changing climate, abiotic stresses become one of the most critical factors that severely reduce crop productivity. For instance, environmental stresses lead to altered nutrient acquisition and biosynthetic capacity that can inhibit plant growth. Furthermore, under abiotic stresses, modification of signaling pathways, expression of genes, proteins, and post-translation may occur, which activates numerous stress-responsive transcription factors to adapt the downstream responses needed to support an effective defense to specific abiotic stress challenges [3,4].

The existence of aerobic organisms, such as plants, depends on oxygen for energy generation systems. Moreover, plants also produce O_2_ during the photosynthesis. Molecular oxygen could be excited, forming reactive oxygen species (ROS). This ROS includes singlet oxygen (^1^O_2_), superoxide radical (O_2_^•−^), hydroxyl radical (^•^OH), and hydrogen peroxide (H_2_O_2_) [5,6]. The generation of toxic ROS as a by-product occurs in various cellular sites, such as mitochondria, chloroplast, peroxisome, and apoplast [7]. Under normal conditions, a balance exists between the production and scavenging of ROS by the coordinated action of antioxidant defense system components within the plants [8]. The generation of ROS at a lower level during normal growing conditions takes part in crucial intra- and inter-cellular signaling processes for inducing a positive response in antioxidant defense systems and the biological processes such as cellular proliferation, differentiation, or stress adaptation mechanisms [5].

On the other hand, disproportionation between ROS generation and scavenging leads to oxidative damage under stress conditions hampering normal cellular function, damaging biomolecules such as carbohydrates, lipids, proteins, and DNA, and finally causes cell death [8]. Thus, a stringent regulation between ROS production and scavenging that aids a plant in utilizing ROS as a defense molecule under stress conditions is crucial [9]. To regulate cell redox homeostasis under a stressful situation, plants utilize a multifaceted and strong antioxidant defense system where non-enzymatic and enzymatic components perform their function in sensing and elimination/detoxification of excess ROS [10]. Therefore, different plants have varied capacities to tolerate oxidative stress that depends on the ability of their antioxidant machinery. Moreover, ROS induced redox signals regulate various vital processes of the cellular environment. For example, ROS-induced redox signaling in plants induces programs of gene expression and aids in maintaining cellular redox homeostasis.

Moreover, the progression of different basic biological processes related to cellular differentiation, proliferation, and cell death are also attributed to ROS-induced redox signaling. Previously, it was thought that these biological processes are attributed to ROS-induced oxidative damage; but recent reports suggest that these processes are triggered by the ROS activating programmed cell death pathway [11]. Henceforth, it is evident that ROS-induced redox signaling plays a vital function in the various physiological responses.

Therefore, this review provides an in-depth look at recent findings related to ROS metabolism domains in plants such as ROS production sites, antioxidant networks, and their synergistic and counter effects under environmental stresses with a particular focus on oxidative stress.

## 2. Types of Reactive Oxygen Species

Partially reduced or activated molecular O_2_ or the primary product or by-product of O_2_-containing molecules showing higher reactivity than atmospheric O_2_ are ROS [12,13]. The transfer of energy or electrons produces ROS from O_2_ molecules; the most common cellular ROS are H_2_O_2_, O_2_^•−^, ^•^OH, and ^1^O_2_ in plants [13,14].

**Figure 1:**
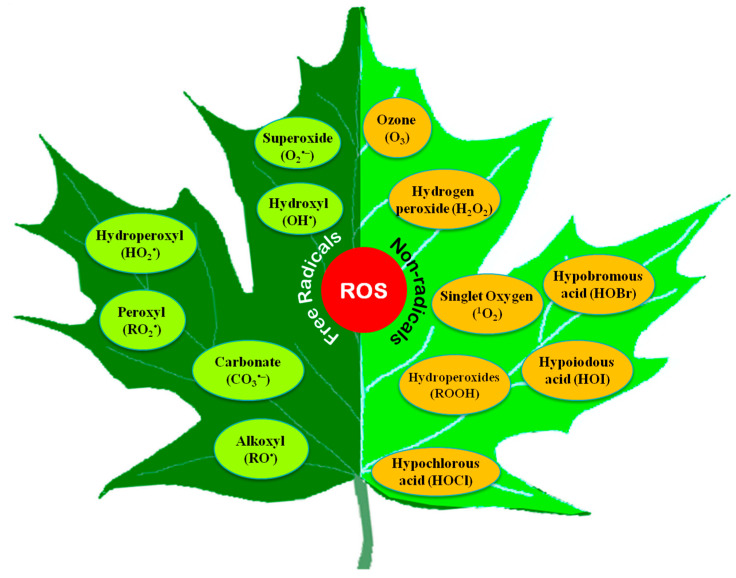
Different reactive oxygen species found in plants.

Both free radical and non-radical ROS are produced in cells (Figure 1). Among them, O_2_^•−^, OH^•^, alkoxyl radical (RO^•^), and peroxyl radical (ROO^•^) are the free radicals, while H_2_O_2_ and ^1^O_2_ are the non-radicals [15]. Some other non-radical ROS, such as hypochlorous acid (HOCl), and excited carbonyl (RO*), are also found in plants [16]. Moreover, some acidic compounds (hypochlorous acid, HOCl; hypoiodous acid, HOI; and hypobromous acid, HOBr) and some radical compounds like carbonate (CO_3_^•−^) are also incorporated into ROS [17].

Besides, peroxyl radical (LOO^•^), alkoxyl radical (LO^•^), hydroperoxyl radical (HO_2_^•^), peroxynitrite (HNO_3_^−^), ozone (O_3_), and trichloromethyl peroxyl radical (Cl_3_COO^•^) could also be found in biological systems.

## 3. Generation of ROS in Plant Cell

**Figure 2:**
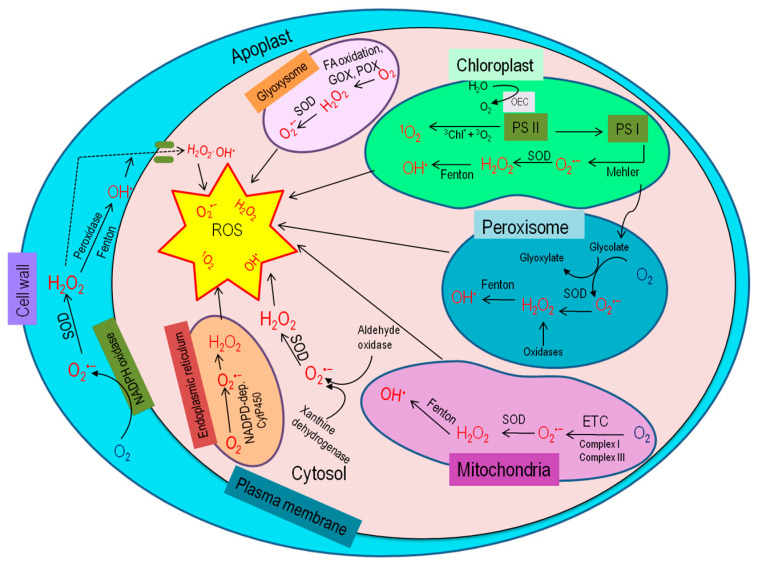
Site and generation of ROS within the plant cell [16].

Under stress conditions, the elevation of ROS relies on subcellular ROS metabolism and their transformation from one to another, which varies for different ROS types, cellular compartments, and even cell types (Figure 2). The diffusion distance of different ROS is also different; but it is to be noted that H_2_O_2_ may be one that is capable of moving from one cell compartment (or even cell) to another [18,19,20]. Therefore, cellular ROS accumulation also depends on the compartmental specific signaling effect and ROS detoxification [21,22].

To better understand ROS scavenging tactics, it is necessary to know the subcellular compartment-specific ROS generation. Different cell compartments have their specific ROS generation and detoxification capacity, being of vital importance for regulating ROS scavenging systems and keeping the ROS below the threshold level for protecting cellular components or initiating signaling cascade.

### 3.1. Chloroplast

The chloroplast is one of the leading ROS production sites in plants [23,24], where ROS generation, directly and indirectly, depends on the interaction of chlorophyll (chl) and light. Here, triplet chl and electron transport chain (ETC), specifically PSI and PSII, are the primary sources for ROS production [25,26,27]. In PSI, O_2_^•−^ is produced by Mehler reaction and then superoxide dismutase (SOD) converts them into H_2_O_2_ [26]. In the presence of metal ions such as Fe^2+^, more highly reactive short-lived HO^•^ are formed from O_2_^•−^ and H_2_O_2_ [27,28]. Environmental stresses cause stomatal closure, and consequent lowering of CO_2_ level provokes the chloroplastic ROS production [29].

### 3.2. Peroxisome

Abiotic stresses induce higher photorespiration in the peroxisome. Here, glycolate oxidase (GOX) is the crucial player for elevated ROS production [30]. In the peroxisome, O_2_^•−^ can be produced at the organelle matrix by xanthine oxidase (XOD) and peroxisomal membrane NADPH oxidase [31]. Metalloenzymes, SODs, later dismutate O_2_^•−^ into H_2_O_2_. Different types of SODs, including Cu-Zn-SOD and Mn-SOD, have been discovered in peroxisomes of various plant species [31]. Peroxisome is considered one of the major sites for H_2_O_2_ generation [31,32]. Besides, photorespiration GOX reaction, O_2_^•−^ disproportionation, β-oxidation of fatty acids, flavin oxidase polyamine oxidase, sulfite oxidase, copper amine oxidase, and sarcosine oxidase activity are the prime metabolic processes through which H_2_O_2_ are produced in peroxisomes [16,30,33].

### 3.3. Mitochondria

In the non-green plant organs, particularly in the root, mitochondria are the major ROS generation sites [26]. Mitochondrial ETC holds electrons with sufficient free energy, from which 0.2–2% of electrons transferred interact with O_2_ to produce ROS, and hence, apparently 1–2% of O_2_ becomes partially reduced [23,24,34]. In such a case, complex I and III in the ETC of mitochondria are the two major sites for ROS production [35]. On abiotic stresses, excessive amounts of ROS are accumulated through electron leakage from both complexes I and III, and produce O_2_^•−^, which is later catalyzed by Mn-SOD and Cu-Zn-SOD and produce H_2_O_2_ [27,36].

### 3.4. Cell Wall

Complex structures made by poly-saccharides, phenolics, and proteins in the plant cell walls are the active site for ROS production [37]. Stressed-plants become prone to growth reduction by differential cell wall growth, where ROS, along with peroxidase, triggers polymerization of glycoproteins and phenolic compounds to make cell walls rigid [24,38,39]. These cell wall-associated peroxidases catalyze H_2_O_2_ generation in the presence of NADH, where the NADH is solely provided from malate dehydrogenase [24]. Additionally, diamine oxidases cause ROS generation in the cell wall by reducing diamines or polyamines to quinine [40]. Under stress conditions, lipoxygenase (LOX)–induced polyunsaturated fatty acid (PUFA) hydroperoxidation is another potential source of ROS [41].

### 3.5. Plasma Membrane

In the plasma membrane, O_2_^•−^ generation is mediated by NADPH oxidase and quinine reductase [42], where electron transportation from cytoplasmic NADPH is catalyzed by NADPH oxidase to form O_2_^•−^, which is again converted into H_2_O_2_.

Reactive oxygen species are also produced in the endoplasmic reticulum, glyoxysome, and cytosol [43]. In the endoplasmic reticulum, Cyt P450 produces O_2_^•−^ as a by-product during reaction with an organic substrate to form a free radical intermediate (Cyt P450-ROO^−^), where NADPH is the electron donor [40]. In glyoxysome, both O_2_^•−^ and H_2_O_2_ are produced from the fatty acid oxidation by GOX and urate oxidase activities [44]. Moreover, both XOD and aldehyde oxidase (AO) are actively engaged in ROS production in the cytosol [43,45].

## 4. Outcome and Indicators of Oxidative Stress

**Figure 3:**
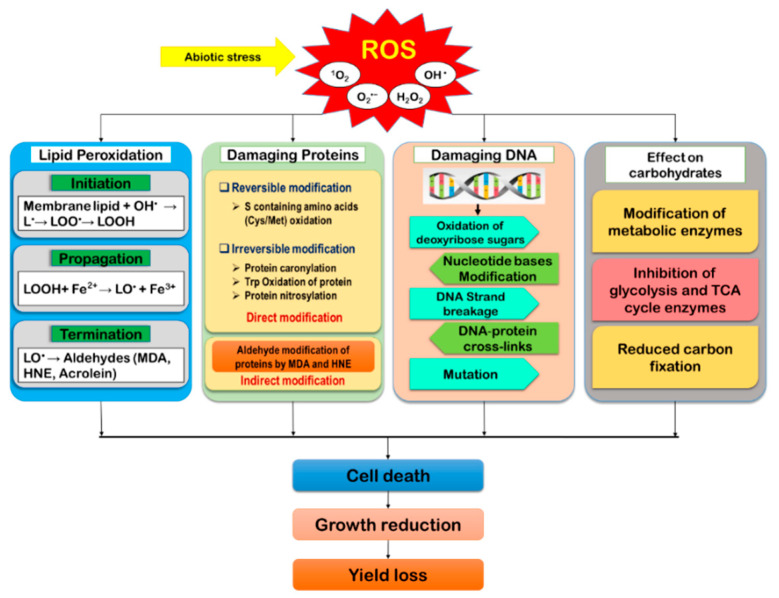
Reactive oxygen species-mediated cellular damage and its consequences in plants. (Lipid free radical, L^•^; lipid peroxyl radical, LOO^•^; lipid hydroperoxide, LOOH; lipid alkoxyl radical, LO^•^).

As the by-products of aerobic metabolism, ROS are found to generate naturally in plants. However, when exposed to a stressful environment, the over-generation of ROS disrupts the equilibrium between ROS accumulation and scavenging, causing oxidative damage of biomolecules that cause cellular damage and death [16,46]. Under oxidative stress, biomolecules like lipids, proteins, DNA, carbohydrates, polynucleic acids, et cetera, become reversibly or irreversibly modified (Figure 3).

### 4.1. Damage to Lipids

Under oxidative stress, over-accumulated ROS causes lipid peroxidation (LPO), causing chain reactions and creates free radicals, resulting in lipid denaturation [47]. In the membrane phospholipids, the most susceptible sites for ROS attack are the carbon (C) atoms and the ester linkage between fatty acids and glycerol. Moreover, ^1^O_2_ and OH^•^ attack the plasma membrane polyunsaturated fatty acids (linoleic and linolenic acids) [48]. Lipid peroxidation is divided into three definite consecutive stages, including (i) initiation, (ii) propagation/progression, and (iii) termination. Of all three steps, initiation is the rate-limiting step. In this process, H^+^ is subtracted from the methylene group (-CH_2_), giving rise to a carbon-centered radical -^•^CH- or lipid-free radical (L^•^). Due to H^+^ subtraction, the double bond in the fatty acid weakens, facilitating the C-H bond. Thus, the lipids remain vulnerable to free radicals and peroxidation [49]. In the propagation stage, L^•^ activates O_2_, generating the lipid peroxyl radical (LOO^•^), and the abstracting of a second H^+^ from nearby fatty acid produces the lipid hydroperoxide (LOOH) and causes another L^•^ [50]. Subsequently, the LOOH is cleaved by the reduced transition metals like Fe^2+^ or Cu^+^, forming a highly reactive radical called the lipid alkoxyl (LO^•^) and resulting in the formation of different aldehydes, alkanes, lipid epoxides, and so on. The termination of the LPO occurs through the formation of various lipid dimmers from lipids-derived radicals.

L-H + OH^•^ → L^•^ (lipid radical) + H_2_O

L^•^ + O_2_ → LOO^•^ (lipid peroxyl radical)

LOO^•^+ L-H → LOOH (lipid hydroperoxide) + L^•^

LOOH + Fe^2+^ → LO^•^ (lipid alkoxyl radical) + Fe^3+^

Singlet oxygen evolved in the PSII reaction center, further reacts with the lipid double bonds forming LOOH [49]. Moreover, ^1^O_2_ can be produced from the reaction of two LOO^•^ damaging membranes’ fluidity, collapsing membrane function, and causing the oxidation and dysfunctioning of membrane proteins, receptors, and localized enzymes, respectively [51]. Furthermore, by damaging membranes, severe LPO collapses their barrier function; thus, the cellular organelles disintegrate. Besides, LPO causes oxidative malfunctions of proteins, DNA, and RNA together with forming some disrupted and altered aldehyde substances like malondialdehyde (MDA), acrolein, and 4-hydroxy-2-nonenal (HNE), which are the oxidative stress markers in plants [49,52].

### 4.2. Damage to Cellular Proteins

Reactive oxygen species or other by-products of oxidative stress alter the covalent bond and induce protein oxidation. Any proteinogenic amino acid can be oxidized by ROS, altering the metabolic, structural, transport, or regulatory activities of proteins. As a secondary effect, some lipid peroxidation products like hydroxynonenal and MDA can react with proteins that modify arginine (Arg), lysine (Lys), proline (Pro), threonine (Thr), and tryptophan (Trp) and result in proteolytic degradation. These proteinogenic amino acids give rise to free carbonyl groups and become more susceptible to proteolytic activity [49]. Essential plant protein complexes including PSI protein complex (PSI-A to PSI-O and LHCI (Lhca1, Lhca2, Lhca3, Lhca4)), D1 protein of PSII, ribulose-1,5-bisphosphate carboxylase/oxygenase (RuBisCo), and SOD require the presence of ^•^OH and ^1^O_2_ for oxidative damages, where the study revealed that ^1^O_2_ is specifically found to affect D1 protein; whereas, increased activity of catalytically-active transition metal (^•^OH) is required for the damages of the others [53]. Collectively, ROS (^•^OH, ^1^O_2_, and H_2_O_2_) result in the oxidation of side chain residues of amino acids, protein-protein cross-linkages formation, protein backbone oxidation, as well as protein fragmentation [48]. Reactive oxygen species (^•^OH, ^1^O_2_) mainly target the proteins having sulfur-containing amino acids and thiol groups. The cysteine (Cys) and methionine (Met) modified residues containing thiol and sulfur groups are inevitably attacked by ^•^OH and ^1^O_2_. Oxidative damages of proteins are mostly irreversible except those with S-containing amino acids (Met and Cys). The ^•^OH and ^1^O_2_ form oxidized derivatives of amino acids sequentially (cysteine → cystine → cysteine sulfenic acid → cysteine sulfinic acid → cysteic acid) [52,54]. Therefore, disulfide bond formation, Cys oxidation, nitrosylation, glutathionylation, and sulfhydration result from redox modification of proteins. Furthermore, Kale et al. [55] proposed that, the formation of HO^•^ at Mn_4_O_5_Ca cluster and the nonheme iron of PSII resulted in the oxidation of specific amino acid residues of the proteins (D1, D2). Besides, oxidative modification of D1:^130^E and D2:^246^M residues were also evident by the formation of O_2_^•−^ (by reducing O_2_). Again, several native amino acid residues were found to be oxidized by ROS; for example, D1, D2, and CP43 subunits in the locality of the cluster Mn_4_O_5_Ca, a peptide of the D1 protein (^130^E–^136^R), and some other oxidized amino residues in the location of PheoD1 and Q_A_ were demonstrated in several studies [56,57,58].

### 4.3. Damage to Nucleotides and DNA

The main damaging factor for polynucleic acids are the ^•^OH, which alters nucleotide bases (purine and pyrimidine) by abstracting H^+^ from the C-H bonds of 2-deoxyribose and methyl group, causing deoxyribose radical, hydroxyl methyl urea, thymine glycol, et cetera, and breaks the double-stranded DNA into single-strands. Damage to DNA due to oxidative stress has been reported by several researchers [59,60]. Furthermore, ROS also damages DNA nucleotide by oxidizing deoxyribose sugar, modifying nucleotide bases, abstracting nucleotides, and DNA protein cross-linking. As a consequence of base oxidation, some damaging products (8-hydroxyquinine, dehydro-2′-deoxyguanosine, etc.) are formed, which cause irreparable cross-links and those are very lethal to plant cells [61]. If these damages are not repaired before the next replication or transcription, DNA denaturation and unfolding are the obvious results [52]. Additionally, incorrect protein sequences are also found as important consequences. Damage of DNA affects plant growth and development, directly affecting various physiological processes; for example, abnormal synthesis of protein and damage of photosynthetic proteins, et cetera. It can also arrest transcription, signal transduction, replication errors, and whole genomic instability [20]. Besides, DNA bases are not always damaged by direct oxidation but also through the reactive intermediates (generated from ROS attack) reacting with the macromolecules. For instance, polyunsaturated fatty acid residues of membrane phospholipids are often attacked by oxygen radicals and are considered vital indirect oxidative damage. Malondialdehyde, acrolein, and crotonaldehyde are some reactive by-products of membrane LPO [62].

### 4.4. Effect on Carbohydrates

In plants, under oxidative stress, redox modification of glycolysis and TCA cycle enzymes are among the primary damaging responses. With an increased ROS level, pentose phosphate pathway enzymes, for example, glyceraldehyde 3-phosphate dehydrogenase and fructose-1,6-bisphosphate aldolase, are inhibited. To cope with the high ROS level, these inhibited enzymes in the pentose phosphate pathway increase the carbon flux (by increasing cycle metabolites, ribose 5-P, and ribulose 5-P intermediates) to produce necessary NADPH [63]. Besides, enzymes of the TCA cycle are severely affected during oxidative stresses, and also inhibit enzyme aconitase and accelerate citrate biosynthesis. Contrarily, citrate accumulation plays vital roles as an introducer of alternative oxidase ROS detoxification under stress [64]. Lehman et al. [63] also demonstrated that under oxidative stress, reduced glycolysis and metabolism of the TCA cycle and amino acid have resulted in the plant. In this study, C flow in *Arabidopsis* root was investigated, kinetic analysis of ^13^C-Glc showed a decreased labeling for citrate, isocitrate, fumarate, malate, succinate, and 2-oxoglutarate, which suggested an inhibited C flux throughout the TCA cycle.

## 5. Oxidative Stress under Abiotic Stress

### 5.1. Drought

Drought stress induces stomatal closure and reduces CO_2_ fixation in plant leaves. Consequently, this stress creates disequilibrium between light capturing and utilization; as a result, the photosynthetic rate is reduced. During drought, photochemistry of chloroplasts become altered, and an imbalance between the electron release and acceptance results in the increased generation of ROS from the excess light energy in the photosystems. In fact, the absorbed light energy that cannot go to CO_2_ fixation, produce ROS [16,46]. Drought stress-induced H_2_O_2_ generation directly results from photorespiration [32]. Additionally, under drought stress, if chloroplast are exposed to excess light energy, ferredoxin becomes highly reduced; the regeneration of NADP^+^ is hindered, which interferes with the acceptance of electrons causing reduced ETC as well as greater electron leakage, which contribute in overproducing ROS [65,66,67]. Drought-induced major and obvious oxidative stress markers are LPO (MDA) and H_2_O_2_ accumulation causing the dysfunctioning of various cellular and physiological processes, including stomatal conductance, membrane functions, water-use efficiency, carboxylation efficiency, respiration, photosynthesis, transpiration, and so on [68]. Reports indicate increased MDA and H_2_O_2_ contents under drought stress in many plant species, for example, rapeseed, maize, soybean, alfalfa, chili, et cetera, which together with the other toxic ROS gives rise to oxidative damages (Table 1).

Performance of *Vigna radiata* L. plants were studied under drought stress (5% polyethylene glycol, PEG; 48 h), singly or together with high temperature (HT) stress and then compared with the control grown plants. Drought and HT either singly or in combination caused higher generation of ROS including free radicals and non-radicals (H_2_O_2_ and O_2_^•−^) along with the enhanced activity of the oxidative enzyme (LOX) resulting in increased LPO indicated by higher MDA levels in *V. radiata* L., compared to the control [69]. Hasanuzzaman et al. [66,70] also documented increased MDA and H_2_O_2_ content in *Brassica napus* L. cv. Bina Sharisha-3 under drought stresses (10 and 20% PEG), where the addition of PEG-6000 in the growth medium resulted in osmotic stress. Nahar et al. [71] found overgeneration of toxic free radicals O_2_^•^^−^ with increased LPO and thiobarbituric acid reactive substances (TBARS) in *Oryza sativa* L. plants when subjected to drought (15 and 20% PEG) for seven days. Sarker and Oba [72] revealed higher electrolyte leakage (EL) resulting from increasing the drought stress severity in *Amaranthus tricolor* plants. In another study, declined EL level by 11, 26, and 47% under mild to severe levels of drought stresses induced by 5, 10, and 15% PEG (3 w), respectively, were reported in *Glycine max* [73]. *Triticum aestivum* L. subjected to severe drought stress (70% FC, field capacity) showed maximum TBARS, EL, and H_2_O_2_ contents of 31, 25, and 38%, respectively, compared to controls [74]. Hussain et al. [75] also demonstrated similar oxidative stress in drought-affected *Zea mays* L. when investigating the effects of drought stress (50% FC, 15 d) on two-hybrid maize varieties, and found an overproduction of toxic ROS (O_2_^•−^, H_2_O_2,_ and ^•^OH) and enhanced accumulation of MDA leading to oxidative stress condition. While working with *O. sativa* L. subsp. japonica. cv. Nipponbare; drought stress (20% PEG) increased O_2_^•−^ by 23%, enhanced H_2_O_2_ content by 1.21-fold, and increased MDA content by 16%, compared to controls, which were liable for creating oxidative damage [76]. Interestingly, drought-induced higher oxidative stress intensities varied among the cultivars and tolerant lentil cultivar (PDL-2) accumulated less ROS and reduced oxidative damage and showed better performance, compared to the sensitive cultivar (JL-3) under drought stress [77]. From these reports, it is obvious that drought stress induces oxidative stress in plants via enhancing toxic ROS levels that are deleterious for vital processes in plants.

### 5.2. Salinity

Salinity restricts crop productivity, particularly in the arid and semi-arid regions as well as in coastal soils. Salinity poses osmotic stress, ion toxicity, genotoxicity, nutritional deficiency, as well as initiates overproduction of ROS, leading to oxidative stress [78]. Under salinity stress, the root tissues suffer the most, followed by mature and young leaves, which are the least affected. Both mild (75 mM NaCl) and severe (150 mM NaCl) doses of salinity caused the increase in MDA, H_2_O_2_, and EL of roots and mature leaf pairs of maize plants, including higher damage severity found under severe stress [79]. Using comet assay, Saha et al. [59] studied the oxidative DNA damage of mung bean under salt stress. They observed salinity-induced enhanced damage of DNA in seven day old mung bean seedlings, which was correlated to oxidative stress. They also found accelerated ROS accumulation in a dose-dependent manner causing larger DNA damage. Salt tolerant *T. aestivum* cv. BARI Gom-28 showed higher H_2_O_2_ and MDA content by 230 and 61% where 41 and 90%, respectively, were found in sensitive cultivars [80]. Although higher H_2_O_2_ was observed in tolerant plants along with lower cellular damage than sensitive plants and these higher H_2_O_2_ might be performing signaling roles. It can be suggested that salt-sensitive cultivars suffered more from oxidative stress. Two-fold higher ROS generation (O_2_^•−^ and H_2_O_2_) along with higher MDA and EL were observed in mung beans under 100 mM NaCl and thus depicted increased oxidative damage [47]. Salt stress caused two times higher ROS generation with elevated LPO and EL in rice roots [81]. Similarly, salinity-mediated elevation of oxidative stress markers including O_2_^•−^, H_2_O_2_, EL, and MDA by 157, 176, 158, and 94%, respectively, observed in tomatoes [82]. From these reports, it is obvious that salinity causes oxidative stress which is lethal for vital processes in plants.

### 5.3. Metals/Metalloids Toxicity

Metals/metalloids toxicity interrupts not only morpho-physiological traits but also causes enhanced oxidative stress resulting from lack of balance between antioxidant defense system and ROS production [83,84,85,86,87,88,89]. El-Amier et al. [90] reported increased levels of LPO and H_2_O_2_ accumulation in Ni (100 µM) stressed *Pisum sativum* L. seedlings. Such increments of oxidative stress indicators was even higher in the same crop with the same concentration of Cd as well. Meanwhile, Cd stress raised the MDA and H_2_O_2_ production in different crops including *V. radiata* L. [83], *B. napus* L. [85], *B. juncea* L. [89], *A. thaliana* [91], and *Cucumis sativus* [92] under different levels of stress. Apart from MDA and H_2_O_2_, the rate of O_2_^•−^ production was also higher in *V. radiata* L. [83]. Another study demonstrated the oxidative damages under Pb stress conditions in wheat plants [86]. Nahar et al. [84] reported higher levels of H_2_O_2_, O_2_^•−^, and MDA contents, and LOX activity by 83, 110, 97 and 72%, respectively, in *V. radiata* L. cv. BARI Mung-2 when exposed to Al stress (0.5 mM) for 48 h. *B. juncea* seedlings exposed to Cr stress (0.15 and 0.3 mM, 5 d) exhibited higher TBARS and H_2_O_2_ contents as well as LOX than that of the control plants [88]. Thus, it is clear that metals/metalloids toxicity increased oxidative stress as depicted by oxidative stress markers such as MDA and H_2_O_2_.

### 5.4. High Temperature

When plants are exposed to HT, heat inactivation occurs on both sides of the electron acceptor and donor in PSII. On the PSII electron donor side, heat inactivation is linked with the inhibition of oxygen-evolving complex (OEC) through removing the extrinsic proteins from their binding sites via the release of Ca^2+^ and Mn^2+^, which is required for H_2_O splitting [93,94,95]. On the PSII electron acceptor side, heat inactivation impaired the electron flow from Q_A_ to Q_B_ due to the increased redox potential of Q_A_/Q_A_^•−^ [95]. This impairs electron flow, which can exert damaging effects on the PSII reaction centers, including D1 and D2 proteins; causing further damage to D1 protein generating ^1^O_2_ on the PSII electron acceptor side [95]. On the electron donor side of PSII, due to incomplete oxidation of H_2_O, H_2_O_2_ forms, after that it has been converted into ^•^OH radicals by Fenton reaction [95]. Heat inactivation also occurred due to the moderate temperature in spinach thylakoids causing LPO and damage to D1 and LHCII as well [96]. Moreover, it has been suggested that sink capacity declines due to the slowing down of carbon fixation, resulting in an increased excitation pressure in the chloroplasts. In PSII, this high excitation pressure causes photoinhibition [97]. High temperature also affects the reaction center of PSI resulting in photoinhibition, which is a rare case in nature due to the P700 oxidation system of the plant that can suppress the excessive excitation pressure of P700 [98]. Several studies reported that unlike PSII, PSI is not a limiting factor in the overall photosynthetic activity of plants [93,99,100]. High temperature (35/32 °C day/night) stress slowed down the electron flow to the PSII reaction center and reduced quantum efficiency (Fv/Fm) and down-regulated photochemistry of PSII of rice (cv. IR64 and Huanghuazhan) [101]. However, short-lived and unstable ^1^O_2_ has a great impact on photosynthesis once it is formed. Moreover, due to instability and higher oxidation-reduction activity, O_2_^•−^ is a vital precursor for various ROS products. In a study, 21-d-old purslane (*Portulaca oleracea* L.) seedlings were treated with HT (42 °C, 7 d) showed higher levels of O_2_^•−^, EL, and MDA content by 2.4, 3.84-fold, and 23%, respectively, than that of controls [102].

Awasthi et al. [103] tested HT stress (32/20 °C day/night, 7 d) in heat-tolerant and heat-sensitive chickpea plants and observed 6.5-fold increased H_2_O_2_ content in leaves of sensitive genotypes than in tolerant ones (5.7-fold). In contrast, MDA content and EL increased by 2.9–6.2-fold and 1.2–1.8-fold in heat-tolerant and sensitive plants, respectively. Furthermore, HT (45/30 ± 2 °C) at three reproductive phases of the cotton plant caused 0.78 times increased MDA content than controls, which affected the cell organelles [104]. Contrarily, Liu et al. [76] found no substantial change in the content of O_2_^•−^ and MDA in rice seeds but H_2_O_2_ increased by 1.27-fold under HT stress (38 °C, 5 d) compared to controls. From these reports, it is clear that HT stress causes oxidative stress as depicted by the oxidative stress markers.

### 5.5. Low Temperature

In several plant species, low temperature (LT) also increases ROS and induces oxidative stress. For example, Guo et al. [105] exposed two sweet sorghum inbred lines (*Sorghum bicolor* L. cv. M81-E and Roma) to LT (10 °C; 0, 12, 24, 36, and 48 h) and observed that MDA content increased to a maximum in both M81-E and Roma by 266% after 48 h of LT stress. Zhang et al. [106] studied two rice cultivars, japonica (Nipponbare) and indica (93-11), under LT stress (2 ± 1 °C; 10, 33, 57 h), where a higher level of H_2_O_2_ at 33 h of stress imposition was reported in cv. Nipponbare, compared to cv. 93-11. A similar trend for H_2_O_2_ overgeneration was also reported by Diao et al. [107] and Ghanbari and Sayyari [108] in tomatoes under LT stress (4 °C, 24 h; and 3 °C, 6 h and 6 d). In another study, LT stressed (12 °C, 6 d) rice seedlings showed a 180% increased MDA and 49% EL, compared to controls [109]. However, it was interestingly seen by Jan et al. [110] that MDA content increased by 16.79% after 24 h, whereas it decreased by 12.21% after 48 h in tomato plants; where EL also showed a similar trend. To evaluate the LT tolerance correlation with the presence of oxidative stress markers in rice shoots and roots, Hsu and Hsu [111] experimented with eight Taiwan rice cultivars exposed to LT (15 °C for 4 d) in a programmable incubator. They observed that the higher growth rate of the LT treated cultivars had a higher level of H_2_O_2_ in their shoots than the roots. In comparison, the LT treated slow-growing cultivars had higher levels of H_2_O_2_ along with higher MDA and EL in their roots than the shoots that caused the interruption of the nutrient uptake from root to shoot. It suggested the presence of higher H_2_O_2_ content correlated positive growth in the shoots but negative in the roots during LT stress (Table 1).

### 5.6. Waterlogging/Flooding

In the natural ecosystem, sudden extreme climate change events like a flood can hamper the natural distribution of plants or even cause extinction [112,113]. Besides causing hypoxia; flooding or waterlogging (WL) may also result in anoxia, which hampers respiration and generates toxic compounds causing impaired metabolic processes [113,114,115]. Ceased growth and biomass production, disturbance in the light interception and root hydraulic conductivity, limitation of stomatal conductance and CO_2_ assimilation, reduced photosynthesis and respiration, and altered accumulation of the secondary metabolites are the main reasons for yield reduction [116]. Such impairments of metabolic processes result in ROS generation and oxidative damages under WL condition.

Studies have revealed flooding-induced increments of LPO, ROS overgeneration, and other oxidative damages in different crops. Some cereal crops, for example, *Z. mays* L., *S. bicolor* L., *Hordeum vulgare* L., et cetera, showed a remarkable increase in LPO and accumulation of H_2_O_2_, O_2_^•−^, and ^•^OH under different durations of WL condition [117,118,119]. Li et al. [117] chose eighteen maize genotypes to evaluate the WL (2 d)-induced changes in LPO. They observed that MDA content increased in only in four genotypes, compared to control plants, but declined in other genotypes, which might be due to the scavenging of ROS by antioxidant enzymes. Two barley (WL sensitive cv. TF57 and WL tolerant cv. TF58) cultivars exposed to WL condition for 21 days resulted in a higher increase of both O_2_^•−^ and MDA contents in susceptible TF57 cultivar, compared to the tolerant TF58 (Table 1; [118]).

### 5.7. High Light

The primary energy source for plants is light. Still, high light (HL) impairs the photosynthetic ETC in PSII, which may lead to the production of ^1^O_2_, resulting in a delayed recovery period of D1 in the PSII core [120]. Furthermore, during electron transfer to O_2_, O_2_^•−^ can be produced at PSI by Mehler reaction or at PSII through Q_A_ to Q_B_ pathway, and H_2_O_2_ can be formed by the univalent reduction of O_2_ at ETC in the plant cell (Table 1; [7]).

According to Awad et al. [121], *A. thaliana* double mutants deficient in two plastids 2-Cys PRXs (2-Cys PRX A and B, *2cpa 2cpb*) and triple mutants deficient in 2-Cys PRXs and *t*APX (*2cpa 2cpb tapx*) leads to accumulation of O_2_^•−^ and H_2_O_2_ and causes photo-bleaching of leaf tissue in HL stress. *Iris pumila* grown in full sunlight had 20% higher MDA content than those grown in shade [122]. Shengxin et al. [123] also noted increased MDA, O_2_^•−^, and H_2_O_2_ values in rapeseed (*B. napus* L. cv. Zhongshuang11) seedlings under HL (550 ± 20 µmol photons m^−2^ s^−1^, 16 h). In contrast, Lima et al. [124] found no significant change in MDA and H_2_O_2_ in 55-d-old cashew plants (*Anacardium occidentale* L.) treated with water deficit followed by HL (850 µmol photons m^−2^ s^−1^, 5 d) showing resistance against water deficit and HT stresses. Recently, Zha et al. [125] divided lettuce plants (*Lactuca sativa* L. cv. ”Yidali”) into three groups of light intensity such as low light (LL; 100 µmol m^−2^ s^−1^), medium light (ML; 200 µmol m^−2^ s^−1^), and high light (HL; 300 µmol m^−2^ s^−1^). They observed that the H_2_O_2_ content increased in lettuce leaves at ML and HL, whereas O_2_^•−^ content increased only in the HL. These increased levels of H_2_O_2_ and O_2_^•−^ contents were seen during the first 6 days of treatment but decreased at 9 days with increased trends found again at 12 days of treatment. The MDA content showed a similar trend to O_2_^•−^ content. In contrast, it was also reported that the H_2_O_2_ and O_2_^•−^ contents remained relatively constant, respectively, at LL stress on the first 9 days of treatment and again increased at 12 days as seen in HL lettuce leaves. All these reports show that HL stress enhanced oxidative stress as depicted by oxidative stress markers. At the same time, the resistant plant genotypes are less susceptible to oxidative damage, compared to sensitive genotypes.

### 5.8. Oxidative Stress under UV-Radiation

The UV radiation (200–400 nm) is detrimental to nucleotides and proteins; consequently, exposure to this radiation causes excess ROS production in plants [126]. Considerable damage to proteins and membranes exerts inhibiting effects on the functioning of mitochondria and chloroplasts, resulting in ROS production [127]. Apart from that, reduction in other plant metabolic functions like CO_2_ assimilation, stomatal conductance, electron transport, and net photosynthesis may also account for the production of ROS in plants exposed to UV radiation [128]. Different experiments have been conducted to understand the UV-radiation-induced oxidative damages, including higher H_2_O_2_ and O_2_^•−^ generation, MDA content, and EL. Tripathi et al. [129] observed increased contents of O_2_^•−^, H_2_O_2_, MDA, and higher EL in *T. aestivum* L. seedlings exposed to two levels of UV-B radiation: ambient (8.6 kJ m^−2^ d^−1^) and enhanced (ambient + 2.8 kJ m^−2^ d^−1^). *G. max* L. plants grown under UV-C light with 0.284 mW cm^−2^ intensity (20 min d^−1^) resulted in remarkably higher O_2_^•−^, H_2_O_2,_ and MDA contents [130]. Four hours of UV-B radiation causes higher levels of EL, MDA, and O_2_^•−^ contents in *Morus alba* seedlings under dark conditions [131]. Enhanced ROS and oxidative stress biomarkers reveal that UV-radiation poses an oxidative stress condition.

### 5.9. Elevated Ozone

Stomata, a crucial interface for gas exchange between plants and the atmosphere, are reported to be affected by the O_3_ concentration. O_3_ imposes phytotoxic impacts on plants via entering through stomata. Exposure to elevated levels of ozone induces oxidative stresses in plants via dissolving entered O_3_ in the aqueous phase of substomatal cavity producing excessive ROS beyond the scavenging capacity of a plant’s intrinsic defense machinery [132]. Depending on the concentration and environmental situations, O_3_ affects the plants to different degrees by causing specific biochemical and molecular responses [133]. For plants acclimated to O_3_ stress, O_3_ signals can initiate PCD during biotic and abiotic stress conditions [134].

The sudden reaction of O_3_ with membrane fatty acids motivates peroxidative processes [135]. In pomegranate, O_3_ exposure caused a 10% increase in O_2_^•−^, 225% in H_2_O_2_, and MDA by 2-fold, compared to controls [136]. Dolker and Agarwal [137] demonstrated a significant elevation in MDA content in *Ischaemum rugosum* Salisb and *Malvastrum coromandelianum* L. under elevated O_3_ exposure of nine months. In wheat, exposure to O_3_ showed an increase in H_2_O_2_, O_2_^•−^, OH^•^ and MDA levels [138]. Lee et al. [139] reported an increase in MDA under O_3_ (86 and 56%) stress compared to controls at 7 and 14 days after exposure, respectively. A similar trend was shown for O_3_ exposure regarding H_2_O_2_, O_2_^•−^, and ^•^OH [139]. Ozone-induced oxidative stress is also associated with the alteration in gas exchange, photosynthetic efficiency, and water relations [140,141,142].

### 5.10. Soil Acidity and Alkalinity

Under extreme pH conditions, the plasma membrane proton pumps try to combat the stress by influx and efflux of H^+^. Still, pH alteration and the excitation pressure lead to the toxic free radical generation together with the severe disruption of cellular and enzymatic activity failures [143]. For instance, Bhuyan et al. [144] investigated the effects of extreme acidic pH (3.5) on wheat (*T. aestivum* L. cvs. BARI Gom-21, 24, 25, 26, and 30) genotypes and increased H_2_O_2_ content together with increased LPO and LOX activity in all cultivars was found. Similarly, Liu et al. [145] found increased cell membrane injury and MDA content in *Medicago sativa* L. cv. Gongnong No. 1, under alkaline stress (pH 11.2). Later on, Bhuyan et al. [146] studied *T. aestivum* L. cv. BARI Gom-25 with both extremely acidic (pH 4.0) and alkaline (pH 8.5) pH and found increased levels of MDA and H_2_O_2_ and LOX activity pointed out oxidative stress under extreme pH. Therefore, the reviewed research findings presented in this section indicate that soil acidity and alkalinity stresses increase oxidative damage in plants.

In addition, acidity stress accelerated toxic metals/metalloids (Fe, Cu, Mn, Zn, and Al) toxicity, together with essential nutrients (P, Mg, Ca, K, and Na due to substantial replacement of cations for H^+^) deficiency and considered as a major limiting factor for plant growth in acid soils [143,147,148]. Similarly, alkalinity stress creates P, Fe, Zn, Mn, Cu, Mo, and B deficiencies. Contrarily, alkaline soils are characterized by B, Na, and Cl toxicities [149]. Nutrient deficiency and metals/metalloids toxicity induced oxidative stress is well studied [150,151,152,153,154,155,156,157]. Acidity induced N, P, K, Ca, Mg, and S deficiency as well as Fe, Mn, B, Zn, and Cu excess was found, with increased ROS level and oxidative stress as well as disrupted redox balance and antioxidant defense [144,158]. Therefore, it could be assumed that acidity or alkalinity induced nutrient deficiency and the metals/metalloids toxicity might be one of the causes for oxidative stress in plants.

On the other hand, plant cells require cytoplasmic pH 7.0–7.5 to maintain the normal physiological activities [159]. It was reported that a single unit decrease of external growing media pH reduces 0.1 units of the cytoplasmic pH [160]. Similarly, increases in external growing media pH causes precipitation of P and other metal ions, consequently increases the absorption of inorganic anions, and disrupts the ion balance [161]. Therefore, both acidic and alkaline pH of growing media alter pH homeostasis, inactivate enzymes, and overgenerate ROS and creates oxidative stress and are considered as the major limiting factors for plant growth [148].

### 5.11. Herbicides Toxicity

Herbicides are often used in cultivated crop plants to easily control weeds. Still, the unconscious use of herbicides may cause oxidative stress in plants. Herbicides increase oxidative stress by overproducing ROS, which destroys plant cell membranes, lipids, photosynthetic pigments, and enzyme activities; therefore, they affect plant growth and productivity (Table 1). Herbicide glyphosate caused oxidative stress in plants by restricting the shikimate pathway, which leads to overproduction of ROS, which disrupted redox homeostasis [6,162]. The application of glyphosate significantly inhibited the growth of *H. vulgare* L. in response to the higher accumulation of H_2_O_2_ (82% in leaves and 123% in roots) and O_2_^•−^, which increased the LPO (MDA; 45% in leaves and 104% in roots) [162]. Glyphosate application in tomato also increased H_2_O_2_ and O_2_^•−^ by 40 and 100%, respectively [6]. Liu et al. [163] observed the increased MDA content with increasing the concentration of picloram in *Eupatorium adenophorum*. Oxidative stress is also observed in various plants after the application of paraquat. Oxidative stress indicators such as MDA, H_2_O_2_, and O_2_^•−^ considerably increased by the application of paraquat in mustard [164]. Besides, the application of the herbicide 2,4-D and its formulation imposed oxidative stress by increasing XOD and LOX activity in pea plants [165] and concomitant increase in MDA content in *M. aquaticum* plants [166].

Alves et al. [167] applied different doses of fomesafen and sulfentrazone in *Raphanus sativus*, *Avena sativa*, *Lupinus albus*, and *V. sativa*, and, where a higher generation of TBARS and altered activity of CAT, APX, and GPX indicate the oxidative stress. Among different species, *V. sativa*, *R. sativus*, and *L. albus* showed a higher damaging effect from sulfentrazone (1.2 kg ha^−1^). Effect of different herbicide applications such as 2,4-D, metsulfuron, metribuzin, iodosulfuron, clodinafop, and bentazon, were studied in the wheat plant. Photosynthesis, transpiration rate, and stomatal conductance were reduced under herbicide toxicity. Lipid peroxidation, CAT activity, and phenols contents were higher, while chl and carotenoids were lower in herbicide stressed wheat plants [168]. Due to the toxic effect of different herbicides, including oxyfluorfen, oxyfluorfen, and pendimethalin, rice plants showed phytotoxicity with reduced height, altered metabolism, accumulation of ROS, and alteration of non-enzymatic and enzymatic components of antioxidant defense machinery. Reduction in photosynthetic pigments, Pro accumulation, protein content, photosynthesis rate, and efficiency of carboxylation, as well as excessive generation of LPO was noticeable as an outcome of the phytotoxic effect of herbicides [169]. A substantial increase in MDA and activity of GR and SOD indicated the paraquat-induced oxidative damage in soybean plants [170].

**Table 1:** Examples of oxidative stress in plants under various environmental stresses.

Plant Species	Stress Levels	Oxidative Stress Indicators	Reference
Drought			
*Lolium perenne* L.	Drought stress, withholding irrigation, 45 d	Increased EL.	[171]
		MDA and H_2_O_2_ content increased.	
*Lens culinaris* Medik. cv. JL-3	Seedlings were exposed to dry air for 4 h, 3 d	Reduced membrane stability index by 57%. MDA content increased by 36%.	[77]
*Arabidopsis thaliana* L.	Drought stress (300 mM d-mannitol), 10 d	Accelerated oxidative stress through elevated ROS generation.	[172]
*Brassica napus* L.	Water deficit (60% FC), 21 d	The LPO product MDA is markedly enhanced.	[173]
		H_2_O_2_ contents remained unchanged.	
*Olea europaea* L.	Water deficit condition by withholding water, 20 d	Increased cell membrane permeability.	[174]
*Oryza sativa* L.	Osmotic stress (15% followed by 20% PEG), 7 d	Higher accumulation of O_2_^•^^−^.	[71]
		Increased LPO as well as TBARS content.	
**Salinity**			
*Triticum aestivum* L.	NaCl (150 mM), 7 d	The H_2_O_2_ content increased by 41%, while MDA content increased by 61% in the salt-tolerant cultivar.	[80]
		The H_2_O_2_ content increased by 230% and MDA content increased by 90% in the salt-sensitive cultivar.	
*Zea mays* L.	NaCl stress; 75 mM (mild) and 150 mM (severe), 3 weeks	Mild and severe stress resulted in a 1.5- and 3-folds increase in H_2_O_2_ in roots.	[79]
		EL and MDA contents also increased similarly.	
*L. culinaris* Medik.	NaCl (100 mM), 3 d	Enhanced H_2_O_2_ and MDA and content by 37 and 139%, respectively, compared to control.	[175]
**Metals/Metalloids Toxicity**			
*Pisum sativum* L.	NiCl_2_ (100 µM), 3 d	Higher content of MDA by almost 4.5-fold and H_2_O_2_ by 7-fold.	[90]
*Withania somnifera* L.	Cadmium sulphate (5, 10, 20, 50, 100, 150, 200 and 300 μM)	Increased MDA content by 2.4-fold at 10 μM cadmium sulfate.	[176]
		Total ROS, H_2_O_2_, O_2_^•−^ and ^•^OH radicals were maximum at 100 μM dose by about 2.1–3.0 -fold than control.	
*O. sativa* L.	CdCl_2_ (2.0 mM), 72 h	Higher MDA and H_2_O_2_ accumulation by 124 and 19%, respectively.	[177]
		LOX activity increased by 114% while shoot EL was 391% higher.	
*Morus alba* L.	PbCl_2_ and CdCl_2_ (100 and 200 μM)	Higher accumulation of H_2_O_2_, O_2_^•−^, MDA and EL were comparably higher intensity in all these under Cd stress than Pb.	[14]
*Cucumis sativus* L. cv. Jingyan-4	Cu^2+^ (80 mM as CuSO_4_), 14 d	Elevation in O_2_^•^^−^, H_2_O_2,_ and ^•^OH accumulation with a higher MDA level.	[178]
**High Temperature**			
*Gossypium hirsutum* L. (84-S and M-503)	30–45 °C, 7 d	MDA content increased by 79% in 84-S and did not change in M-503.	[179]
*Portulaca oleracea* L.	42 °C, 7 d	Increased O_2_^•−^, EL, and MDA contents by 2.4, 3.84-fold, and 23%, respectively.	[102]
*C. sativus* L.	35 ± 1 °C, 7 d	Increased MDA content (60.6%) and O_2_^•−^ (79.9%).	[180]
*Nicotiana tabacum* cv. Bright-Yellow 2	50 °C, 5 min	Increased O_2_^•−^ by 50%.	[181]
		Increased MDA and H_2_O_2_ contents.	
**Low Temperature**			
*O. sativa* cv. Nipponbare and 93–11	2 ± 1 °C, 10, 33, 57 h	H_2_O_2_ (brown spots of histochemical analysis of H_2_O_2_) increased.	[106]
*Calendula officinalis* L.	4 °C for 24, 48, 72, 96 and 120 h	Elevated MDA content (16.79%) and EL (11.78%).	[110]
*O. sativa* cv. Taiwan	15 °C for 4 d	Higher levels of H_2_O_2_ along with MDA in roots decreased the growth rate	[111]
*Prunus armenica* L.	Freezing stress (−3 and −1 °C), 30 min	Increased LPO level, H_2_O_2_ content, and ion leakage percentage	[182]
**Waterlogging/Flooding**			
*Z. mays* L.	Waterlogging, 14 d	Accumulation of MDA, H_2_O_2_, O_2_^•−^ and ^•^OH was increased in WL treatment.	[119]
*P.persica* L. Batsch	Waterlogging, 72 h	H_2_O_2_, O_2_^•−^ accumulation, and cell death intensity increased compared to control plants.	[183]
*G. max* L.	Waterlogging, 10 d	Increased H_2_O_2_, O_2_^•−^ and MDA contents.	[184]
*P. mahaleb* *P. pseudocerasus*, *P. cerasus* × *P. canescens*	Waterlogging, 24 h	Increased MDA, H_2_O_2,_ and O_2_^•−^ accumulation. *P. mahaleb* accumulated much higher MDA, H_2_O_2,_ and O_2_^•−^ than the other two.	[185]
		About 2.2, 7.2, and 1.5-fold higher MDA, H_2_O_2,_ and O_2_^•−^ contents were noticed in stressed *P. mahaleb* than control.	
*C. sativus* L.	Waterlogging, 96 h	Increased H_2_O_2_ and O_2_^•−^ accumulation.	[186]
**High Light**			
*A. thaliana* L.	1000 µmol photons m^−2^ s^−1^, 2 d	^1^O_2_ and H_2_O_2_ increased.	[187]
*O. sativa* L.	1400–1600 µmol photons m^−2^ s^−1^, 1 h	Increase of O_2_^•−^ and H_2_O_2_ in midvein by 1.23 and 1.72-fold, respectively.	[188]
		NADPH/NADP^+^ ratio (2.19-fold) also found higher in midvein.	
*Coffea arabica* L.	1000 µmol photons m^−2^ s^−1^, 12 months	NADPH/NADP^+^ ratio (0.6-fold) lower in HL than low light (1.1 to 1.2-fold).	[135]
*Solanumlycopersicum* L.	500, 1000 µmol photons m^−2^ s^−1^, 5 d	MDA and H_2_O_2_ contents progressively increased by 90 and 83%, respectively.	[189]
**UV-Radiation**			
*T. aestivum* L.	UV-B radiation of 8.6 kJ m^−2^ d^−1^ at 12th and 14th day after emergence	The rate of O_2_^•−^ generation increased by 127%, and the contents of MDA and H_2_O_2_ increased by 64 and 44%, respectively.	[129]
*O. europaea* L. cv. Galega Vulgar	UV-B radiation of 6.5 kJ m^−2^ d^−1^ (UV-B_1_) and 12.4 kJ m^−2^ d^−1^ (UV-B_2_) for 5 d	Almost similar H_2_O_2_ contents with a free radical scavenging capacity—ABTS being higher than the control (UV-B_1_: 23.5% and UV-B_2_: 21.7%).	[190]
**Elevated Ozone**			
*G. max* L.	80 ppb, 6 h d^−1^ for 5 d	TBARS content was higher in saplings of Tracajá cultivar of soybean than in Sambaíba.	[191]
		Plants of both cultivars showed a 2-fold increase in TBARS content than plants maintained under filtered air.	
*S.tuberosum* L.	70 ppb O_3_; 3 months	MDA and H_2_O_2_ increased by 2-fold and 1.5-fold, respectively, at 60 d after emergence.	[192]
*N. tabacum* L. *G. max* L., and *Populus* *tremula* L.	96, 74, and 64 ppb	Increase of MDA content by 97.0, 65.3, and 63.4, respectively in tobacco, soybean, and poplar, respectively.	[193]
		Increased O_2_^•−^ content in poplar (by 18.4%), tobacco (by 18.8%), and soybean (by 45.6%).	
		Increased H_2_O_2_ content of tobacco and soybean by 26.2 and 82.0%, respectively, whereas had no effect on poplar.	
*O. sativa* L.	70–150 ppb for 10 d	Increased MDA content, compared to control.	[194]
*T. aestivum* L.	59.6 ppb; 122 d	MDA content increased in HD2967.	[195]
**Acidity and Alkalinity**			
*O. sativa* L.	Simulated acid rain stress (pH 2.0 or 3.0, 4.0)	The H_2_O_2_ content in the root increased with the decrease of the pH (3.0 or 2.0).	[196]
		Decreased antioxidant enzyme activities. Increased cellular damages.	
*S. lycopersicum* L. cv. Micro-Tom	Simulated acid rain stress (pH 2.5 and 5.6), 17 d	Overaccumulation of ROS.	[197]
		Damaged grana lamella of the chloroplast.	
		Increase of MDA and H_2_O_2_ contents by 63 and 45%, respectively, compared to control.	
*Medicago sativa* L. cv. Gongnong No. 1	Alkaline stress (25 mM Na_2_CO_3_, pH 11.2), 48 h	Increased accumulation of ROS as well as increased oxidative damage.	[145]
		Increased cell membrane injury by 463%. Enhanced MDA content by 57%.	
*Z. mays* L.	Alkaline stress (100 mM and 150 mM Na_2_CO_3_ solution), 10 d	The H_2_O_2_ production increased considerably by 96 and 154% with 100 and 150 mM Na_2_CO_3_ treatments, respectively.	[198]
		Amplified LOX activity by 99 and 167%, in both alkaline stresses, respectively.	
*B. oleracea* L. cv ‘Bronco’	Alkaline stress (50 mM NaHCO_3_: Na_2_CO_3_), pH 9, 25 d	Greater contents of MDA and higher LOX activity.	[199]
		Increased level of ROS specially amplified O_2_^•^^−^ content.	
*O. sativa* L.	Simulated acid rain (SAR) stress (pH 5.5, 5.0, 4.5, 4.0, 3.5, 3.0 or 2.5), 5 d	Overaccumulation of ROS exceeded the scavenging ability of the antioxidant enzymes.	[200]
		Disrupted membrane permeability.	
		Elevated level of H_2_O_2_, O_2_^•^^−^ and MDA, contents by 107, 155 and 187% respectively, were found under the acid rain stress (pH 2.5) over the control.	
**Herbicides Toxicity**			
*Hordeum vulgare* L.	Glyphosate (6 mM)	Increased lipid peroxidation (MDA; 45% in leaves and 104% in roots) and H_2_O_2_ (82% in leaves and 123% in roots), and O_2_^•−^ generation.	[162]
*Salvinia natans* L.	Glyphosate (0.006, 0.03, 0.15, 0.3 and 0.45 mM)	Enhanced MDA and H_2_O_2_ production.	[201]
*S. lycopersicum* L.	Glyphosate (2, 4 and 6 mM)	Higher H_2_O_2_ (40%), and O_2_^•−^ (100%) contents in root at maximum concentration.	[6]
*B. napus* L.	Paraquat (62.5, 125 and 250 mM)	Increased lipid peroxidation (MDA; 24, 71, and 85%), ROS generation (H_2_O_2_; 30, 90, and 134% and O_2_^•−^; 28, 59, and 82%) and LOX activity (69, 167, and 234%).	[164]
*Cucurbita* spp.	Paraquat (0.05, 0.1, 0.2, 0.3 and 0.5 mM)	Increased cellular leakage and MDA production.	[202]
*N. tabacum* cv. oriental	Imazapic (0.03, 0.06 and 0.12 mM)	Increased MDA content.	[203]
*Eupatorium adenophorum*	Picloram (0.1, 0.2, 0.5, 1.0 and 2.0 mM)	Increased EL (32, 36, 42, 43, and 44%) and MDA content (2.23, 2.27, 2.62, 2.71, and 2.93 times).	[163]

## 6. Overview of Plant Antioxidant Defense System

**Figure 4:**
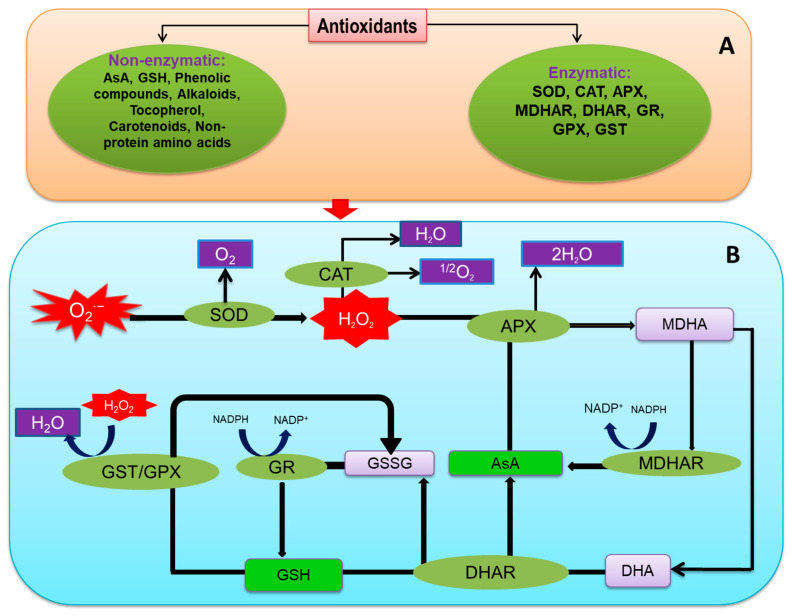
Outline of antioxidant defense mechanisms in plants; (**A**) types of antioxidants and (**B**) mechanism of antioxidant enzymes and low molecular weight antioxidants to detoxify ROS. Additional details are in the text.

Antioxidants are considered vital components for scavenging ROS, which play a critical role in abiotic stresses [204]. Plants have developed a multifaceted antioxidant defense network to reduce ROS overgeneration under different abiotic stresses (Figure 4; [205]). The antioxidant defense system consists of several antioxidants of low molecular weight (AsA, ascorbate; GSH, glutathione; non-protein amino acids; phenolic compounds; α-tocopherol; and some alkaloids) and antioxidant enzymes (SOD; CAT, catalase; POX, peroxidases; APX, ascorbate peroxidase; MDHAR, monodehydroascorbate reductase; DHAR, dehydroascorbate reductase; GR, glutathione reductase; GPX, glutathione peroxidase; GST, glutathione *S*-transferase).

### 6.1. Low Molecular Weight Antioxidants

#### 6.1.1. Ascorbic Acid

Ascorbic acid (vitamin C) is a strong water-soluble antioxidant, abundant in the active growing parts such as meristems, photosynthetic cells, root tips, flowers, and young fruits [206]. Having the potential to donate electrons as a co-enzyme, the AsA participates significantly in scavenging ROS upon stresses [46]. Ascorbate also participates in regenerating α-tocopherol from tocopherol radical scavenging O_2_^•−^ and ^•^OH. The growth and development of plants are considerably influenced by AsA production under abiotic stresses [207]. By regulating cellular water status, AsA improved enzymatic detoxification of ROS (H_2_O_2_) to protect the cells. In plants, many phytohormone biosynthesis pathways are regulated by AsA [208]. Therefore, the exogenous application of AsA increased plant growth by regulating hormonal balance and ion homeostasis [209]. Reports suggested that exogenous AsA application maintained the growth and biochemical processes of cauliflower [210], wheat [211], cucumber [207], canola [212], soybean [213], rapeseed [214], and grapes [215].

#### 6.1.2. Glutathione

As a low molecular weight antioxidant and a non-protein thiol, GSH plays a critical function in regulating intracellular defense by scavenging ROS. Besides, GSH maintains redox homeostasis as a component of the AsA-GSH cycle [46]. It also plays vital roles in detoxification of xenobiotics, signal transduction, transportation of sulfate, and metabolites conjugation [216]. Glutathione also detoxifies atmospheric pollutants such as O_3_ and NO_2_. It also actively participates in recycling the AsA and α-tocopherol [217]. Upon stress, the frequent stimulation of GSH indicated the possible role of regulating defense mechanisms [218].

#### 6.1.3. Tocopherol

The antioxidant tocopherol is found as alpha (α), beta (β), gamma (γ), and delta (δ) forms; mostly synthesized in photosynthetic organs. They protect the photosynthetic membrane by scavenging ROS, mainly ^1^O_2_ and ^•^OH [219].

#### 6.1.4. Carotenoids

The carotenoids mainly accomplish three important activities: (i) absorbing light spectra (between 400 and 550 nm wavelengths), (ii) scavenging harmful ROS during photosynthesis, and (iii) protecting the complex light-harvesting proteins as well as stabilizing thylakoid membranes [220,221]. Carotenoids have a polyene backbone in their structure, which consists of a series of C=C bonds. This particular characteristic is mainly responsible for pigmentation and ROS quenching ability [222].

#### 6.1.5. Flavonoids

Flavonoids are low molecular weight and contain hydroxylation patterns in their molecular structure, indicating the antioxidant capacity [223]. Flavonoids decrease cell damage in plants by scavenging free radicals and protecting cell membranes from LPO [224]. The genes related to flavonoid biosynthesis are highly expressed under stress conditions; therefore, activating defense mechanisms by increasing flavonoid levels. Apart from the antioxidant activity, flavonoids can regulate auxin transport in vivo and give photoprotection. Moreover, flavonoids protect plants against UV light damage by absorbing UV radiation and act as sunscreens. Although light is essential for flavonoid biosynthesis, exposure to UV radiation induces higher levels of flavonoids in plants, which further act in the ROS removal mechanism in plants [225].

### 6.2. Antioxidant Enzymes

#### 6.2.1. Superoxide Dismutase (EC 1.15.1.1)

Superoxide dismutase is a metalloenzyme that shows the frontline defense under excessive ROS generation. In most plant cells, the available SOD concentration is ~10^−5^ M [226]. Based on metal co-factor at active sites, three main SOD types are described—Cu/Zn-SOD, Mn-SOD, and Fe-SOD. In the antioxidant defense network, O_2_^•−^ is dismutased by SOD into H_2_O_2_, this also reduces the option of ^•^OH generation via Haber–Weiss reaction (Figure 4; [226]).

#### 6.2.2. Catalases (EC 1.11.1.6)

The tetrameric haem-containing CAT enzyme rapidly decomposes H_2_O_2_, producing H_2_O and O_2_. All aerobic organisms contain CAT, a unique enzyme for ROS detoxification without any reducing equivalent [227]. Among the antioxidant enzymes CAT possesses, the maximum turnover rate and 26 million H_2_O_2_ molecules can be converted by one CAT molecule in one minute [217]. CAT activity is found in peroxisomes, mitochondria, and cytosol [228].

#### 6.2.3. Peroxidases (EC. 1.11.1.7)

Peroxidases are glycoproteins containing a polypeptide chain with 300–350 amino acid residues. The POX contains three domains—among which a proximal heme-binding domain and a distal heme-binding domain is identified, but the other one is still unknown [229]. Peroxidase mainly oxidizes phenolic compounds (PhOH) and produces phenoxyl radical (PhO^•^), where H_2_O_2_ contributes to this reaction as an electron acceptor, and it is converted to 2H_2_O.

#### 6.2.4. Ascorbate Peroxidase (EC 1.11.1.1)

Another class I heme-peroxidase is APX occurring in several isoforms (cAPX, cytosolic APX; mitAPX, mitochondrial APX; chAPX, chloroplastic APX; and microbody (including peroxisomal and glyoxysomal) APX), mAPX; [31]. All the isoforms function to scavenge H_2_O_2_, but the activity stops without the presence of AsA [46]. Within the AsA-GSH cycle, APX participates in detoxifying H_2_O_2_ and oxidizes AsA to produce monodehydroascorbate (MDHA) and subsequent dehydroascorbate (DHA) (Figure 4).

#### 6.2.5. Monodehydroascorbate Reductase (EC 1.6.5.4)

In the AsA-GSH cycle, MDHAR is an NADH or NADPH-dependent flavin adenine dinucleotide enzyme containing a thiol group involved in the phenoxyl radical reduction and AsA regeneration from MDHA [46,220]. MDRAH has several isoforms based on localization. Therefore, MDHAR genes are present in different cell components like mitochondria, chloroplasts, glyoxysomes, peroxisomes, and cytosol [230]. Respective genes and locations are essential to knowing the role of each isoform. One MDHAR gene can produce two isoforms, and MDHAR genes can vary in different plant species (Figure 4; Table 2; [230]).

#### 6.2.6. Dehydroascorbate Reductase (EC.1.8.5.1)

The enzyme DHAR, is monomeric and is included in the GST super-family, plays a vital role to regenerate AsA by an oxidative reaction, where DHA is recycled [220,248]. In this process, GSSG is from the oxidation of GSH. As a result, the enzyme is also called GSH dehydrogenase or GSH:DHA oxidoreductase [249].

#### 6.2.7. Glutathione Reductase (EC 1.6.4.2)

In the AsA-GSH cycle, GR is another vital enzyme for regulating the redox homeostasis, which reduces GSSG to GSH (Figure 4; Table 2; [250]).The reaction catalyzed by GR involves two steps—in the first step, NADPH reduces the flavin moiety, which is oxidized and reduced forming a disulfide bridge, which is redox-active and generates a thiolate anion and cysteine. In the next step, one GSSG moiety binds with cysteine forming a disulfide bond, finally releasing GSH [251].

#### 6.2.8. Glutathione Peroxidases (EC 1.11.1.9)

The enzyme GPX, is the non-heme peroxidase family member, utilizes GSH and thioredoxin, reduces H_2_O_2_, and protects cells from oxidative damage [252]. Eight GPX proteins were identified in *Arabidopsis*, mainly found in the chloroplast, mitochondria, endoplasmic reticulum, and cytosol [253]. The active site of GPX contains a thiol group (Cys residue) that can bind both GSH and thioredoxin, therefore, considered the redox regulating enzyme (Figure 4; [254,255]).

#### 6.2.9. Glutathione *S*-Transferases (EC 2.5.1.18)

Glutathione *S*-transferases are a ubiquitous large enzyme family regulating versatile functions within plants [256]. The enzyme has three superfamilies based on their localization, viz. cytosolic, mitochondrial, and microsomal. The enzyme is classified into various types, for example, phi, tau, lambda, and DHAR are found in plants; where phi and tau are highly responsible for environmental stresses mitigation [257,258]. Moreover, it accelerates the activity of GPX and significantly reduces the reactive electrophile species generation (Figure 4; [232]).

## 7. Antioxidant Metabolism and the Detoxification of ROS under Environmental Stress

### 7.1. Drought Stress

Under drought stress, an adaptive strategy is activating the antioxidant defense system to fight against the oxidative stresses and to develop tolerance against drought in plants [66,70]. The AsA and GSH are the strongest among the non-enzymatic antioxidants, which provide significant protection against drought-induced oxidative stress. In *O. sativa*, upregulation of AsA and GSH under drought stress (PEG, 15 and 20%) reduced oxidative damages. In *B. napus* cv. Bina Sharisha-3, higher AsA content was found under moderate stress level (10% PEG), but not in severe stress (20% PEG) levels. Moreover, in moderate and severe drought-stressed rapeseed seedlings, GSH content increased by 31 and 26%, respectively, compared to controls, whereas 83 and 225% increases in GSSG contents were also documented [66]. Nahar et al. [69] demonstrated reduced AsA and enhanced DHA contents, which ultimately reduced the overall AsA/DHA ratio by 54% in *V. radiata* L. under drought stress. The GSH/GSSG ratio was also reduced, compared to controls in drought exposed *V. radiata* L. seedlings. Elevated antioxidant enzyme activities also contributed to scavenging the toxic free radicals and protected plants from oxidative damage [71]. Hasanuzzaman et al. [66] revealed enzymatic antioxidants; DHAR, MDHAR, GR, and APX, played essential roles along with the non-enzymatic antioxidants (AsA and GSH) to alleviate drought stress-induced oxidative damage in *B.* *napus* L. cv. Bina Sharisha-3, which decreased the MDA and H_2_O_2_ contents. Upon drought (20% PEG) stress, toxic ROS overgeneration decreased by activated antioxidants in *B. rapa* L., where endogenous AsA and GSH levels increased together with a notable (23%) enhancement in APX activity, as well as increased CAT, GPX, and GR, activity by 29, 26, and 81%, respectively, compared to unstressed seedlings [259]. Liu et al. [76] investigated the drought stress (20% PEG-6000, 5 d) effects on *O. sativa* L. subsp. japonica cv. Nipponbare which demonstrated significantly decreased CAT, APX, and SOD activities and remarkably increased peroxidase (POD) activity by 59%. Rezayian et al. [73] studied the effect of mild to severe drought stress (5, 10, and 15% PEG) on *G. max* L. and found increased SOD and POX activities at a high intensity of stress (15% PEG). Still, most uplifted activities of APX, CAT, and POX were found at low intensity of stress (5% PEG). Meanwhile, uplifted tocopherol (by 26, 26, and 21%) and total phenol (51, 32, and 44%) contents were also demonstrated at three levels of PEG-stresses, respectively. In *T. aestivum* L. cv. Sakha-94 enhanced GPX activity but significantly inhibited CAT activity under drought stress, compared to controls [260]. When exposed to drought stress (irrigation at 60% of soil FC) for 20 days, an upregulated antioxidant defense system was also found in *S. lycopersicum* L. In this case, enzymatic antioxidants like APX, CAT, and SOD activities enhanced by 77, 110, and 66% in contrast, and non-enzymatic antioxidants viz. α-tocopherol, GSH, and AsA increased by 103, 93, and 81%, respectively, compared to controls (Table 2; [261]).

### 7.2. Salinity

Variations in antioxidant activities impart differential salt stress tolerance to plants. These variations are present even at the plant organ level. For instance, AbdElgawad et al. [79] studied antioxidant enzyme activity responses in different organs (roots, young leaves, and mature leaves) of salt-stressed maize plants. More specifically, they observed that DHAR and CAT activity increased in all studied organs while APX, SOD, GR, and GST increased in roots following NaCl application. Antioxidant activities of tolerant (BRS Bojuru) and sensitive (BRS Pampa) rice cultivars were significantly different under salt stress, where increased SOD and CAT activities were observed only in tolerant cultivars [262]. Vighi et al. [262] reported that *OsGR2*, *OsGR3*, *OsAPX3,* and *OsSOD3-Cu/Zn* genes were the markers to differentiate between sensitive and tolerant cultivars upon salinity stress. Chung et al. [263] observed that the antioxidant-related genes *GmCATs* and *GmAPX1* responds under salt stress conditions. Compared to the control plants *GmCAT1*, *GmCAT2*, and *GmAPX1* expression was upregulated by 2- to 3-fold, 3- to 4-fold, and 8- to 9-fold, respectively, in soybean plants under salt stress. Such higher salt tolerances of tolerant cultivars are linked to higher antioxidant levels than salt-sensitive cultivars [264].

Various protectants are reported to increase the salinity-mediated oxidative stress tolerance in plants via modulating antioxidant machinery. For example, salicylic acid and Si supplementation improved salt tolerance in wheat and mung bean, respectively, by increasing APX, CAT, and SOD activities that lowered H_2_O_2_ levels, EL, and MDA content [47,265]. Rady et al. [266] reported that pretreatment with *Moringa oleifera* leaf extract (MLE; 6%) better regulated both the non-enzymatic antioxidants (GSH, AsA, and AsA redox) and enzymatic antioxidants (APX, GPX, POD, SOD, and CAT) than Pro (12 mM) pretreatment for tackling salt stress. From the above reports, it is evident that alterations in the antioxidant system impart salinity tolerance in plants.

### 7.3. Metals/Metalloids Toxicity

The tolerance mechanism of plants to metals/metalloids-induced oxidative stress depends on the antioxidant defense capacity. It is positively correlated with the enhancement of the antioxidant enzyme activities and the improved synthesis of non-enzymatic antioxidants. Apart from the role of ROS scavenging, these components, both enzymatic and non-enzymatic, also help in metal chelation to some extent [267,268,269].

El-Amier et al. [90] observed that *P. sativum* L. seedlings when exposed to Ni (100 µM NiCl_2_, 72 h), GSH content was increased linearly up to 48 h and then declined, whereas GSSG content increased up to 36 h and then decreased. The later reduction of GSH and GSSG contents might disrupt the antioxidative system by Ni-toxicity stress [90]. In rice seedlings, 0.25 and 0.5 mM NiSO_4_.7H_2_O enhanced both the GSH and GSSG contents, of which GSSG was further declined. Moreover, decreased AsA and increased DHA contents and ultimately reduced AsA/DHA ratio under Ni-stress was reversed by the exogenous application of Si (0.05 mM Na_2_SiO_3_) that signifies the ability of Si to upregulate the AsA-GSH cycle [87]. Nickel stress also enhanced APX, MDHAR, DHAR, GR, GPX, and SOD activities, which were further upregulated by the supplementation of Si. The LOX activity was also increased under Ni stress, but Si application reduced LOX actions [87]. Almost 3-fold higher AsA, 2-fold higher GSH, and 4.5-fold higher GSSG contents along with increased SOD and CAT activity were observed in *Cajanas cajan* seedlings when exposed to 10 µM of As (as Na_3_AsO_4_) for 5 days [270]. With higher levels of As stress (150 and 300 µM Na_3_AsO_4_, 35 d) increased activities of SOD, CAT and POD were reported in two different cultivars of *Chenopodium quinoa*, and these enzyme activities were further upregulated with dimethylthiourea (5 mM) treatment [271].

### 7.4. High Temperature

The plants typically use antioxidant protection systems to deal with HT stress, which varies among genotypes [68,272]. Mansoor and Naqvi [273] observed the elevated activity of SOD, POD, and APX in all genotypes of mung bean (*V.* *radiata* L.) at 40 °C except CAT. Sarkar et al. [235] observed an increased activity of CAT, GSH, APX, and POX in all tested wheat cultivars at 30 °C. However, all these enzyme activities decreased in heat-sensitive cultivars, compared to tolerant ones at 35 °C. A similar result for enzymatic antioxidants was reported by Ding et al. [180] in cucumber (*C. sativus* L.) seedlings, in which the activity of SOD, CAT, APX, GR, and POD increased by 16.6, 13, 25.2, 14.4, and 35.4%, respectively, under HT stress (35 °C), while GSH down-regulated as a non-enzymatic antioxidant. In contrast, Awasthi et al. [103] found a higher content of GSH in chickpea (*Cicer* *arietinum* L.) plants at HT stress conditions. Djanaguiraman et al. [274] reported that due to the lower activity of CAT and POX during HT stress, SOD activity decreased, and ROS toxicity increased in both pistils and pollen grains of both pearl millet and sorghum plants. However, in cabbage and kale plants, Soengas et al. [275] found a higher activity of SOD, CAT, and GR, compared to controls under HT stress (32 °C), while compared with cabbage, the activity of SOD recorded was higher in kale plants. In a greenhouse experiment, Sarwar et al. [104] exposed cotton plants to HT stress and treated them with growth regulators (water and H_2_O_2_). After the treatment duration, they observed that the H_2_O_2_ treated plants showed higher SOD (0.75, 0.33, and 0.36-fold) and CAT (2.26, 1.19, and 1.31-fold) activity than plants with water under high, medium, and optimum thermal regimes, respectively.

### 7.5. Low Temperature

Numerous studies have revealed that the elevated SOD activity in plants mediates LT tolerance by scavenging H_2_O_2_ [109,276]. Unlike SOD, CAT and APX also catalyze the H_2_O_2_. Wang et al. [277] found the higher activity of CAT in indica 93-11 after 24 h of LT stress, while activity declined with increased stress duration. Shi et al. [278], Wani et al. [279], and Mohammadrezakhani et al. [280] observed that due to the LT stress, CAT activity increased significantly in *Cynodon dactylon*, *Capsella bursa-pastoris,* and *Citrus reticulata*. Whereas, higher APX activity in *Jatropha macrocarpa* even under high H_2_O_2_ levels improved LT stress resistance while *J. curcas* could not withstand LT condition due to the reduced (>6-fold) activity of APX [281]. Cheng et al. [282] kept watermelon (*Citrullus lanatus*) plants at 10/5 °C for 7 days and observed that GSH/GSSG and AsA/DHA ratios were increased by 30.6 and 214.3%, respectively, compared to controls, which is related to antioxidant defense system activation. Han et al. [109] treated 14-day-old rice seedlings at 12 °C for 6 days and reported that the GSH and GSSG contents as well as GSH/GSSG ratio were increased significantly along with higher activities of SOD, POD, and CAT. Chen et al. [283] also reported that at 4 °C, GR, DHAR, and MDHAR activities increased by 20.26, 7.64, and 16.60%, respectively, and AsA, DHA, and GSH also increased by 12.13, 7.89, and 56.09%, respectively, compared to controls, resulting in direct scavenging of ROS. From the above examples, it is obvious that modulation in antioxidant machinery exerts a positive impact in LT tolerance ability.

### 7.6. Waterlogging/Flooding

Several crop species exhibit survival abilities under waterlogged or flooded conditions for short or even longer durations sometimes; rice can be a good example. In addition to formation of aerenchyma and adventitious roots, plants may also exhibit the ability of modulating antioxidant defense systems to combat the waterlogging-induced oxidative damages [116]. Some cereal crops like *Z. mays* L., *S. bicolor* L., and *H. vulgare* L. have been shown to be damaged at the cellular level by higher LPO and ROS and further antioxidative defense response [118,119]. Upregulation or downregulation of these antioxidants has also been reported in *G. max* L. [239,240], *L. esculentum* [284], *Sesamum indicum* L. [285], and *Spinacia oleracea* L. [286]. da-Silva and do Amarante [184] studied WL modulated upregulation of antioxidant defense system in *G. max* L. and observed higher activities of APX, CAT, and SOD in stressed plants along with higher ROS and MDA accumulation. Moreover, reduced AsA with a higher DHA level indicated ROS scavenging by spending AsA [184]. Waterlogging stress also showed significant variation in SOD, CAT, and POD in *B. napus* L., regardless of its growing condition (pot or field), along with higher MDA [287]. Some sensitive and tolerant species of *Prunus* were subjected to WL, and upregulation of antioxidants (AsA and GSH contents; CAT and POD activities) were recorded, which indicated the existence of ROS scavenging systems in tolerant cultivars (*Prunus pseudocerasus* and *P. cerasus* × *P. canescens*) [185].

### 7.7. High Light

While studying the impact of HL, Shengxin et al. [123] disclosed that the activity of SODs, CAT, and POD decreased in rapeseed seedlings under HL conditions. Similarly, Lu et al. [191] observed decreased POD and SOD activity due to HL stress in tomatoes. In contrast, SOD, CAT, APX, and POD activities upregulated in the moderate light treated Chl-8 rice genotype leaves than Zhefu 802 [288]. In HL stress, higher SOD and CAT activity was observed in *pgl* rice genotype compared to Zhefu 802 [289]. Lima et al. [124] demonstrated decreased CAT activity along with upregulated APX and SOD activity in HL exposed (12 h) cashew plants. Furthermore, as total AsA content decreased by 25%, the total GSH content increased by 63% in HL exposed (12 h) cashew plants [124]. Similarly, HL stress down-regulated *APX2*, *DHAR1*, *CDS1*, *CDS2*, and *FDS2* while upregulated *APX1*, *CAT2*, and *FDS1* in *Arabidopsis* mutant (*pgr5*), compared to WT (Table 2; [244]). Flavonoid biosynthesis is light-dependent. Higher light intensities stimulate the flavonoids synthesis in *Passiflora suberosa* L., which protects the plants from HL damage, as documented by Ni et al. [290]. Moreover, Stewart et al. [291] narrated about water-insoluble antioxidant carotenoids (the xanthophylls zeaxanthin and lutein as well as β-carotene), which maintain the higher growth rate in *Lemna gibba* L. even under very HL (700 µmol photons m^−2^ s^−1^, 7 d). It resulted from a combination of the decline in the chl synthesis and higher zeaxanthin accumulation, which restricted the accumulation of excessive energy from excitation.

### 7.8. UV-Radiation

Tripathi et al. [129] exposed *T. aestivum* L. seedlings to two levels of UV-B radiation: ambient (8.6 kJ m^−2^ d^−1^) and enhanced (ambient + 2.8 kJ m^−2^ d^−1^) resulted in upregulated CAT and GPX activity along with reduced SOD and APX activities. Such a reduction in SOD activity was also observed in *A. thaliana* L. (genotype rsr4-1). In contrast, genotype C24 showed increased POD, APX, and GPX activities exposed to UV-B radiation (3.9 kJ m^−2^, 4 d). UV-C radiation-induced enhancement of SOD and POD activities was also reported in *G. max* L. [130]. Seedlings of *G. max* L. grown under UV-C light with 0.284 mW cm^−2^ intensity (20 min d^−1^) resulted in enhanced activity of CAT, SOD, and POD that was further upregulated with exogenous application of AsA (10 mg L^−1^) [130]. In *Olea europaea* L. cv. Galega Vulgar plants, 23.5, and 21.7% higher free radical scavenging capacity (ABTS) was recorded in two levels of UV-B (6.5 and 12.4 kJ m^−2^ d^−1^) treatments, respectively. As a result, the levels of H_2_O_2_ were almost similar to control plants [190]. However, in addition to such antioxidant defense responses, some terrestrial plants have also been documented to synthesize some protective phytochemicals (e.g., AsA, hydroxycinnamates, flavonoids, vitamins, etc.) when exposed to UV-B radiation [126,292,293].

### 7.9. Elevated Ozone

Ozone causes various restrictions in plants such as visible foliage injury, early senescence of leaves, stomatal closure, and inhibition of carbon transport, et cetera. Of these, stomatal closure is considered a direct cause of the yield reduction of plants as it reduces carbon uptake/carbon fixation and ultimately induces reduction in photosynthesis. Hence, decreased transportation of fixed carbon towards edible plant parts, for example, grains result in yield reduction due to low supply of source towards sink [294]. Furthermore, O_3_ directly disturbs the carbon translocation in phloem from leaves to other parts such as roots or edible portions and thus has more influence on yield reduction [295]. Under such scenario, a plant’s ability to withstand O_3_ stress can stabilize yield reductions. In this regard, antioxidants can play a vital role in regulation and detoxification of toxic ROS. The influence of O_3_ in regulating antioxidant activities in two rice cultivars was studied by Tammam et al. [296]. Both rice cultivars showed higher SOD and APX activity in shoots, but GR and GST activities were reduced in both shoots and roots. In a two-year study on rice cultivars with contrasting ability towards O_3_ stress tolerance, Wang et al. [297] demonstrated that CAT, POD, and GPX activities were decreased in O_3_ sensitive cultivars. The authors concluded that O_3_ tolerance improved at different growth stages via regulating antioxidant activities in tolerant cultivars. In another study, O_3_ exposure-induced ROS production was scavenged via regulation of antioxidant activities in wheat leaves [298].

### 7.10. Soil Acidity and Alkalinity

Both acidity and alkalinity stress altered the antioxidant defense system of plants. Uplifted APX, CAT, SOD, and POD activities under acidic pH were found in rice [196] and tomato [197], but Bhuyan et al. [144] found down-regulated SOD and DHAR activities while GR activity remained unchanged in wheat seedlings subjected to acidity stress. Similarly, a higher pH level (alkalinity stress) resulted in lower antioxidant enzyme activities in rice. In contrast, elevated APX, CAT, SOD, and POD activities were increased in *Z. mays* L. under alkaline stress. Moreover, the redox balance of AsA/DHA severely declined. Although GSH content increased, GSSG content decreased, while GPX, GST, and GR activities were also reduced [198]. Later on, Bhuyan et al. [146] observed the affect of acidity (pH 4.0) and alkalinity (pH 8.5)-stresses on *T. aestivum* L. and found decreased AsA and GSH contents, with upregulated APX, CAT, GPX, and GST activities under both acidic and alkaline-stress.

### 7.11. Herbicides

The plants can mitigate herbicide-induced toxicity by two resistance mechanisms. The first one is target-site resistance, in other words, structural modification or activity enhancement of herbicide targeted proteins. The second one is non-target site resistance, in other words, developing the herbicide detoxification system [299]. The herbicide detoxification system contained three enzymatic phases: (i) in phase I, CytP450 monooxygenases; (ii) in phase II, GSH transferases and glycosyl transferases; and (iii) in phase III, tonoplast localized ATP-binding cassette transporters, while GST showed a major role in this system [300]. Several reports proved the antioxidant defense mechanisms in plants to reduce herbicide toxicity. Glyphosate toxicity alleviated in *H. vulgare* by improving the activity of antioxidant enzyme CAT (80% in leaves and 46% in roots), APX (106% in leaves and 97% in roots), and GST (61% in leaves and 95% in roots) [162]. The activity of SOD, POD, CAT, and APX improved in *Salvinia natans*, which reduced glyphosate-induced oxidative stress [201]. In *T. aestivum*, Hasanuzzaman et al. [164] reported increased enzymatic antioxidant activities; and AsA and GSH contents to mitigate paraquat toxicity. Higher GSH content and high CAT, APX, GST, and GR activities were also observed to improve antioxidant defense in imazapic-induced *Nicotiana tabacum* [203].

## 8. Conclusions

It is well established that ROS play a vital role in regulating various responses under both biotic and abiotic stresses in plants. The domain of plant stress is quite vast, and the number of studies grew dramatically over the last 20 years. The interesting phenomenon among all these studies is the dual role of ROS in plants. Firstly, they are unavoidable toxic metabolic by-products; and secondly, they play signaling roles during stress conditions. Although it seems that ROS are damage causing agents in plants, their importance for stimulating the stress signaling component to stop further losses is also notable. Even with the continuous increase of stress-related publications, there exists quite little novelty in the information. Most reports just confirmed the previous results rather than bringing new insights into this domain. Therefore, there should be attempts at new approaches for obtaining the novel aspects regarding ROS metabolism. It is still an open question of how plants feel stressed and get ready for upcoming stress threats. Due to their highly reactive nature and short half-life, further research is needed on ROS chemistry and metabolism. For instance, different aspects related to ROS metabolism regulation, particularly under multiple environmental stresses, which remain unanswered. Therefore, future studies should focus on more polished techniques such as advanced imaging techniques, fluorescence in situ hybridization (FISH), advanced functional genomics, metabolomics, and proteomics to better understand the ROS metabolism in crop plants. Exogenous ROS application at proper dose and duration to improve signaling cascades and subsequent stress tolerance also warrants further research.

## Figures and Tables

**Table 2:** Antioxidant metabolism and defense under various environmental stresses.

Plant Species	Stress Levels	Antioxidant Metabolism	Reference
Drought			
*Lolium perenne* L.	Drought stress (withholding irrigation), 45 d	Significant improvement of APX, CAT, and SOD activity, but POD activity remained unchanged up to 15 d, whereas increased thereafter.	[171]
*Vigna radiata* L. cv. BARI Mung-2	Drought stress (5% PEG), 48 h	Decreased AsA and high DHA content with reduced AsA/DHA ratio.	[69]
		Downregulated GSH/GSSG ratio.	
		Increased GR, APX, GPX, and GST activity with reduced CAT and MDHAR activity.	
*Lens culinaris* L. Cultivars: drought tolerant (PDL-2) and sensitive (JL-3)	Drought stress (seedlings exposed to dry air for 4h), 3 d	Upregulation of SOD, APX, and GPX in both tolerant and sensitive cultivars (higher in PDL-2 by 32, 44, and 57% than in JL-3).	[77]
		CAT activity showed no significant difference.	
*Triticum aestivum* L. cv. Jing 852	Drought stress (10% PEG), 6 h	CAT and SOD activities consistently increased.	[206]
		POD and APX activities were increased initially but declined later.	
*T. aestivum* L.	Drought stress (70, 50, and 35% of soil water holding capacity)	Uplifting SOD and CAT activities in a dose-dependent manner compared to respective control.	[74]
*Brassica napus* L. cvs. Dunkeld and Cyclone	Water deficit (60% FC), 21 d	Slightly enhanced total phenolics in both canola cultivars.	[173]
		Increased activities of POD and CAT enzymes.	
		Dunkeld performed better compared to Cyclone in POD activity, whereas, in the case of SOD activity, Cyclone was better.	
*Sorghum bicolor* L. cvs. M-81E and Roma	Drought stress (sand water content 4.2%), 7 d	APX and SOD activities increased in both cultivars.	[105]
		M-81E had uplifted enzymatic antioxidant activities as well as stronger scavenging ability than Roma.	
*Oryza sativa* L.	Drought stress (15 and 20% PEG), 7 d	Elevated antioxidant enzyme activities, including CAT, SOD, APX, GPX, and GR.	[71]
		Increased level of AsA and GSH.	
*Capsicum annuum* L.	Drought stress (without watering), 7 d	Increased APX and CAT activities.	[231]
		No significant changes in SOD activity were observed.	
*T. aestivum* L. cv. Sakha-94	Drought stress (stopped irrigation at 10 DAS), 11 d	Enhanced GPX but inhibited CAT activity.	[232]
**Salinity**			
*O. sativa* L.	Salinity 150 and 300 mM NaCl, 48 h	In the BRRI dhan54, Pro, GB, and GSH contents increased.	[219]
		In BRRI dhan49, Pro and GB increased together with enhanced SOD activity.	
*V. radiata* L.	Salinity 200 mM NaCl, 48 h	Exogenous application of PAs enhanced AsA content and AsA/DHA while restored CAT activity.	[233]
*V. radiata* L.	Salinity 50–200 mM NaCl, 2 d to 3 weeks	Transgenic plants overexpressing *AtNHX1* from *A. thaliana* enhanced APX, SOD, GPX and GR.	[234]
**High Temperature**			
*Gossypium hirsutum* (84-S and M-503)	30–45 °C, 7 d	Enhanced the activity of FeSOD and Cu/ZnSOD in M-503; also increased APX and GR activities.	[179]
*Portulaca oleracea* L.	42 °C, 7 d	Increased SOD and POD activities except for CAT.	[102]
*T. aestivum* L. cv. Gayetri, Gandhari, Kedar, PBW343	25, 30, 35, and 40 °C, 6 h	Elevated activity of CAT (1.02-fold), POD and APX.	[235]
		GSH content increased.	
**Low Temperature**			
*Ipomoea batatas* L.	4 and 13 °C, 8 weeks	Transgenic plants overexpressing *AtP3B* enhanced POD and CAT activities.	[236]
*Lycopersicon esculentum*	4 °C, 5 d	Transgenic plants overexpressed *AtDREB1A* enhanced SOD and CAT activities.	[237]
*Camellia sinensis* L.	−5 °C, 3, 6, and 12 h	High tea polyphenol to the amino acid ratio by 48, 83, and 86%, respectively.	[99]
*Triticum spp.*	Frost injury (−3, −5, and −7 °C), 24 h	Amplified transcript level of GST and APX enzymes in all wheat cultivars under frost injury.	[238]
**Waterlogging/Flooding**			
*G. max* cv. Daewon	Waterlogging, 2 d	GSH activity was reduced in both shoot and root.	[239]
		GR activity was reduced in shoots but unaffected in the roots.	
*Prunuspersica* L. Batsch	Waterlogging, 72 h	Activities of CAT, SOD, and POD increased up to 24 h but decreased at 48 and 72 h.	[181]
*G. max* L.	Waterlogging, 10 d	Up-stimulation of SOD, CAT, and APX activities.	[182]
*P. mahaleb,* *P. pseudocerasus,* *P. cerasus × P. canescens*	Waterlogging, 24 h	Higher the POD, CAT, and GR activities in all rootstock.	[183]
		AsA and DHA contents increased in *P. cerasus* × *P. canescens* and decreased in the other two species.	
		GSH and GSSG contents decreased in *Prunus mahaleb* while increased significantly in the other two species.	
*G. max* L. genotypes Grobogan, Willis and Detam-1	Inundation condition, 72 h	Activities of both SOD and POD enzymes were higher compared to control plants.	[240]
*Zea mays* L. cvs. Zhengdan-958 and Xing Ken-6	Waterlogging, 14 d	Higher activities of SOD, POD, CAT, APX and GR.	[119]
**Metal/Metalloids Toxicity**			
*Nicotiana tabacum* and *Petunia × atkinsiana*	100 mM of CuSO_4_, ZnSO_4_, K_2_Cr_2_O_7_, or 500 mM MnSO_4_,10 d	Plants overexpressing *RsMYB1* improved the activities of SOD, CAT, POX, and GST.	[241]
*N. tabacum*	100 μM CdCl_2_, 1 mM MnCl_2_, 500 μM ZnSO_4_, or 50 μM CuSO_4_, 72 h	Genotypes overexpressing *LmSAP* improved the activities of SOD, CAT, and POD.	[242]
*O. sativa* L.	CdCl_2_ (2.0 mM), 72 h	Decreased AsA and DHA contents with a sharp increase in both GSH and GSSG contents.	[177]
		Higher activity of APX, MDHAR, GR, SOD, GPX.	
		Reduced the activity of DHAR (by 33%), CAT (by 35%), and GST.	
*Solanumlycopersicum* cv. çiko F1	Cd, Cu, and Pb (50 ppm)	Decreased APX activity in leaves except for Cu-toxicity, which was increased.	[243]
		Increased in POD and SOD activities.	
*Withania somnifera* L.	CdSO_4_ (5 μM, 10 μM, 20 μM, 50 μM, 100 μM, 150 μM, 200 μM and 300 μM)	Tocopherol content was the maximum at 10 μM, about 2.75-fold.	[176]
		GSH content increased by 2.02-fold.	
		AsA and DHA content was enhanced by 4.46-, 2.16- and 38.75-fold, respectively.	
		MDHAR, DHAR, GR, and GPX activity upregulated.	
*Cucumis sativus* L. cv. Jingyan-4	80 mM Cu^2+^ was supplied as CuSO_4,_ 14 d	Decreased SOD, POD, and APX activity in roots but increased in leaves.	[178]
		Increased SOD, POD, APX and GR activities in leaves.	
		Increased GSH and GSSG and their ratio in both leaves and root tissue.	
*Morus alba* L.	PbCl_2_ and CdCl_2_ at 100 and 200 μM, respectively	Lower activity of APX and SOD but slightly increased SOD activity was found only in the lower dose of Pb.	[14]
**High Light**			
*O. sativa* L. cv. *Liangyoupeijiu*	1400–1600 µmol photons m^−2^ s^−1^, 1 h	CAT, DHAR, MDHAR, and POD activity were higher (0.147 to 0.534-fold) in leaf lamina, while SOD and APX were higher in midvein.	[188]
		The AsA and GSH contents increased, and DHA and GSSG decreased.	
		AsA/DHA and GSH/GSSG ratios increased in midvein.	
*S.lycopersicum* L.	500, 1000 µmol photons m^−2^ s^−1^, 5 d	SOD and POD activity down-regulated.	[189]
		APX gene expression was higher, and GR expression was lower.	
*Anacardium occidentale* L.	850 µmol photons m^−2^ s^−1^, 5 d	CAT activity decreased while the activity of APX and SOD upregulated.	[124]
		AsA content decreased by 25%, and GSH content increased by 63%.	
*A. thaliana pgr5* and WT glabrous 1	1000 µmol photons m^−2^ s^−1^, 1 h	CAT activity increased in mutants than WT under control.	[244]
		DHAR activity increased in HL treated mutants. Expression of *APX2*, *DHAR1, CDS1*, *CDS2,* and *FDS2* were down-regulated, and *APX1*, *CAT2,* and *FDS1* were upregulated in the mutant.	
**UV-Radiation**			
*T. aestivum* L. cv. HP 1761	UV-B radiation (8.6 kJ m^−2^ d^−1^) at 12th and 14th day after emergence	Higher accumulation of AsA was recorded.	[129]
		Lower SOD and APX activities were observed, while CAT and GPX activities increased.	
*A. thaliana* cvs. C24 and rsr4-1	UV-B radiation (3.9 kJ m^−2^) up to 4 h d^–1^, 4 d	Activity of SOD was not affected in C24 but drastically reduced in rsr4-1.	[245]
		In C24, the activity of POD, APX and GPX increased while remaining unchanged in rsr4-1.	
*Olea europaea* L. cv. Galega Vulgar	UV-B radiation (6.5 kJ m^−2^ d^−1^, UV-B_1_) and 12.4 kJ m^−2^ d^−1^, UV-B_2_), 5 d	Reduced activities of GR (by 75%) and APX (by 36%) under UV-B_1_ treatment, while GR increased by 59% under UV-B_2_ treatment remaining APX unaffected.	[190]
		Activities of SOD, CAT, and GPX increased in a dose-dependent manner with the highest value of UV-B_2_ treatment.	
*G. max* cv. Jin 36	UV-C radiation (0.284 mW cm^−2^) for 20 min per day, 50 d	Activities of SOD and POD increased by 30 and 28%, respectively.	[130]
**Elevated Ozone**			
*S. tuberosum* L.	70 ppb O_3_, 3 months	POX and APX activity enhanced by 73 and 21%, respectively, under ambient CO_2_ and elevated O_3_.	[192]
		CAT, POX, GR, and SOD activities increased.	
*Malus crabapple*	100 ± 10 nL L^−1^ O_3_, 3 h	Increased CAT, POD, and SOD by 85, 50, and 51%, respectively.	[246]
*O. sativa*	70–150 ppb O_3_, 10 d	Increased AsA level.	[194]
*T. aestivum*	59.6 ppb O_3_, 122 d	Increased CAT, GR, APX, and POD activities.	[195]
**Acidity and Alkalinity**			
*S. lycopersicum* L. cv. Micro-Tom	Simulated acid rain stress (pH 2.5 and 5.6), 17 d	Enhanced activities of antioxidant enzymes (CAT, APX, SOD and POD), increased total phenolic, flavonoids, Pro and total antioxidant contents.	[197]
*Medicago sativa* L. cv. Gongnong No. 1	Alkaline stress (25 mM Na_2_CO_3_, pH 11.2), 48 h	Decreased oxidative stress-induced damages by upregulating the AsA content and POD and CAT activities.	[145]
*B. oleracea* L. cv. Bronco’	Alkaline stress (50 mM NaHCO_3_:Na_2_CO_3_) (pH 9), 25 d	Declined total GSH concentration, GR and POX activity.	[199]
		APX activity increased.	
		Total AsA, reduced AsA and DHA diminished.	
*T.aestivum* L. cv. BARI Gom-25	Extreme acidic (pH 4.0) and extreme alkaline (pH 8.5)-stress, 72 h	Extreme pH levels (4.0, 5.5, and 8.5) decreased AsA and GSH contents.	[146]
		Upregulated activities of CAT, APX, GPX, and GST.	
		DHAR and SOD activity down-regulated under extreme pH stress, compared to control.	
*T.aestivum* L. cvs. BARI Gom-21, 24–26 and 30	Different pH of growth medium, 6.5 (control), 5.5, 4.5 (acidic) and 3.5 (extreme acidic), 4 d	Decreased antioxidant enzyme activity with the gradual increase in the acidity severity in all the cultivars.	[144]
		Decreased GSH and GSSG ratio.	
		Upregulated antioxidant enzymes’ activities, including APX, GPX, GR, MDHAR, DHAR and GST were observed in BARI Gom-26.	
**Herbicides Toxicity**			
*Hordeum vulgare* L.	Glyphosate (6 mM)	Increased the activity of CAT (80% in leaves and 46% in roots), APX (106% in leaves and 97% in roots), and GST (61% in leaves and 95% in roots)	[162]
*Salvinia natans* L.	Glyphosate (0.006, 0.03, 0.15, 0.3 and 0.45 mM)	Increased CAT, SOD, POD and APX activity.	[201]
*B. napus* L.	Paraquat (62.5, 125 and 250 mM)	Increased enzymatic antioxidant activities, and AsA and GSH content.	[164]
*N. tabacum* cv. oriental	Imazapic (0.030, 0.060 and 0.120 mM)	Improved GSH content.	[203]
		Increased CAT, APX, GST and GR activities.	
*Cucurbita* spp.	Paraquat (0.05, 0.1, 0.2, 0.3, 0.5 and 1.0 mM)	Lower MDA content and cellular leakage in youngest leaves (4th leaf) than older leaves.	[202]
		Increased CAT, POX, and APX (2 times) activity in youngest leaves.	
*C. sativus* L.	Paraquat (0.05, 0.1, 0.2, 0.3, 0.5 and 1.0 mM)	Lower LPO and higher antioxidant enzyme activity.	[247]
